# Supplementary material for: Structural and genetic diversity in the secreted mucins MUC5AC and MUC5B
Source: Am J Hum Genet. 2024 Jul 10;111(8):1700–16. doi: 10.1016/j.ajhg.2024.06.007 (PMC11344006; doi:10.1016/j.ajhg.2024.06.007)
Supplement: Document S2. Article plus supplemental information [file mmc3.pdf]

Structural and genetic diversity in the secreted mucins *MUC5AC* and *MUC5B*

Graphical abstract

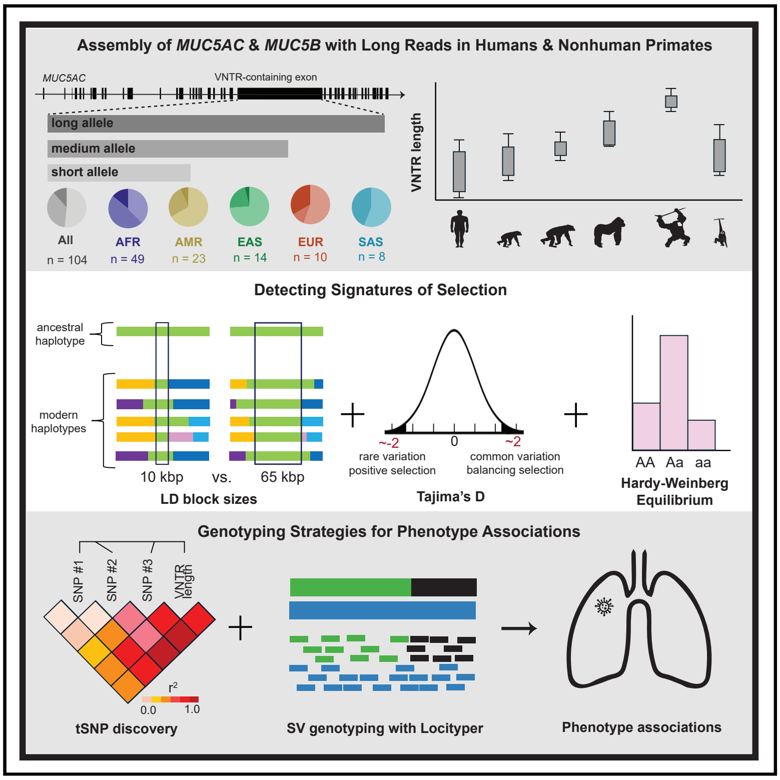

Authors

Elizabeth G. Plender, Timofey Prodanov, PingHsun Hsieh, ..., Tobias Marschall, Jesse D. Bloom, Evan E. Eichler

Correspondence

ee3@uw.edu

***MUC5AC* and *MUC5B* are polymorphic loci that are difficult to sequence due to coding variable number tandem repeats (VNTRs). Using long-read sequencing, we characterized genetic diversity in these genes across human populations, detected signatures of selection, and developed genotyping strategies for disease associations.**

Plender et al., 2024, The American Journal of Human Genetics *111*, 1700–1716
August 8, 2024 © 2024 The Author(s).
https://doi.org/10.1016/j.ajhg.2024.06.007

ARTICLE

Structural and genetic diversity in the secreted mucins *MUC5AC* and *MUC5B*

Elizabeth G. Plender,[1,2] Timofey Prodanov,[3,4] PingHsun Hsieh,[1,5] Evangelos Nizamis,[6] William T. Harvey,[1] Arvis Sulovari,[1,7] Katherine M. Munson,[1] Eli J. Kaufman,[6] Wanda K. O'Neal,[8] Paul N. Valdmanis,[1,6] Tobias Marschall,[3,4] Jesse D. Bloom,[1,2,9] and Evan E. Eichler[1,10,*]

Summary

The secreted mucins MUC5AC and MUC5B are large glycoproteins that play critical defensive roles in pathogen entrapment and mucociliary clearance. Their respective genes contain polymorphic and degenerate protein-coding variable number tandem repeats (VNTRs) that make the loci difficult to investigate with short reads. We characterize the structural diversity of *MUC5AC* and *MUC5B* by long-read sequencing and assembly of 206 human and 20 nonhuman primate (NHP) haplotypes. We find that human *MUC5B* is largely invariant (5,761–5,762 amino acids [aa]); however, seven haplotypes have expanded VNTRs (6,291–7,019 aa). In contrast, 30 allelic variants of *MUC5AC* encode 16 distinct proteins (5,249–6,325 aa) with cysteine-rich domain and VNTR copy-number variation. We group *MUC5AC* alleles into three phylogenetic clades: H1 (46%, ~5,654 aa), H2 (33%, ~5,742 aa), and H3 (7%, ~6,325 aa). The two most common human *MUC5AC* variants are smaller than NHP gene models, suggesting a reduction in protein length during recent human evolution. Linkage disequilibrium and Tajima's D analyses reveal that East Asians carry exceptionally large blocks with an excess of rare variation ($p < 0.05$) at *MUC5AC*. To validate this result, we use Locityper for genotyping *MUC5AC* haplogroups in 2,600 unrelated samples from the 1000 Genomes Project. We observe a signature of positive selection in H1 among East Asians and a depletion of the likely ancestral haplogroup (H3). In Europeans, H3 alleles show an excess of common variation and deviate from Hardy-Weinberg equilibrium ($p < 0.05$), consistent with heterozygote advantage and balancing selection. This study provides a generalizable strategy to characterize complex protein-coding VNTRs for improved disease associations.

Introduction

Mucosal linings serve a dynamic role at the interface between internal tissues and the external environment. In the lumen of the lungs, epithelial cells provide defensive functionalities through mucociliary clearance, a mechanism in which mucus traps inhaled pathogens for mechanical removal.[1] The mucins MUC5AC and MUC5B are major components of mucus that contribute to its barrier function and act as receptor decoys for pathogens, such as the influenza virus that binds directly to mucin sialic acids.[2] These polymeric glycoproteins thus provide a critical innate immunological role in defending the airways against environmental insults; however, they have also been implicated in the pathogenicity of muco-obstructive airway diseases like asthma and cystic fibrosis.[3]

Despite their fundamental roles in maintaining epithelial homeostasis, *MUC5AC* and *MUC5B* sequence variation remains poorly understood. The challenge in assessing these loci is that they harbor large central exons (60%–80% of total coding sequence) composed of variable number tandem repeats (VNTRs). These VNTRs encode numerous serine and threonine residues that are decorated with sialic acid, a terminal sugar moiety that is bound by the glycoproteins of some viral pathogens.[2,4] Limitations of short-read sequencing in assembling these repetitive loci have hindered efforts to accurately resolve copy-number variation.[5,6] VNTR structural variants may affect the functional ability of mucins to act as barriers to pathogens and change their biophysical properties; therefore, it is critical that the sequences of these loci are characterized in many human genomes to discover the common patterns of variation directly affecting protein function.

Long-read sequencing technologies allow for the characterization of *MUC5AC* and *MUC5B* with haplotype-level resolution. Previously, gene references for both loci were constructed using Pacific Biosciences (PacBio) single-molecule, real-time (SMRT) sequencing from a limited number of humans. Four genome assemblies were used to characterize three distinct *MUC5AC* haplotypes for VNTR structural variation.[7] However, analyses of *MUC5AC* allele sizes via Southern blot suggest a much greater extent of human diversity.[8] Many additional human genomes have recently been sequenced with more accurate high-fidelity (HiFi)

[1]Department of Genome Sciences, University of Washington School of Medicine, Seattle, WA 98195, USA; [2]Basic Sciences Division and Computational Biology Program, Fred Hutchinson Cancer Center, Seattle, WA 98109, USA; [3]Institute for Medical Biometry and Bioinformatics, Medical Faculty, Heinrich Heine University, Moorenstr. 5, 40225 Düsseldorf, Germany; [4]Center for Digital Medicine, Heinrich Heine University, Moorenstr. 5, 40225 Düsseldorf, Germany; [5]Department of Genetics, Cell Biology, and Development, University of Minnesota Medical School, Minneapolis, MN 55455, USA; [6]Division of Medical Genetics, University of Washington School of Medicine, Seattle, WA 98195, USA; [7]Computational Biology, Cajal Neuroscience Inc, Seattle, WA 98102, USA; [8]Marsico Lung Institute/UNC CF Research Center, School of Medicine, University of North Carolina at Chapel Hill, Chapel Hill, North Carolina 27599, USA; [9]Howard Hughes Medical Institute, Fred Hutchinson Cancer Center, Seattle, WA 98109, USA; [10]Howard Hughes Medical Institute, University of Washington, Seattle, WA 98195, USA
*Correspondence: ee3@uw.edu
https://doi.org/10.1016/j.ajhg.2024.06.007.
© 2024 The Author(s). This is an open access article under the CC BY license (http://creativecommons.org/licenses/by/4.0/).

circular consensus sequencing (CCS) as part of the Human Genome Structural Variation Consortium (HGSVC)[9] and the Human Pangenome Reference Consortium (HPRC).[10] Here, we leverage the large-scale sequencing efforts of the HGSVC and HPRC to explore common patterns of genetic variation in *MUC5AC* and *MUC5B*, specifically within the VNTR portion of the molecule. Using 206 diverse human haplotypes assembled with high-quality PacBio HiFi CCS reads, we characterize the genetic diversity of these loci in different human populations. We compare the human alleles of *MUC5AC* and *MUC5B* to that of five nonhuman primate (NHP) species (chimpanzee, bonobo, gorilla, orangutan, and gibbon) to distinguish human-specific patterns of variation. Finally, we explore methods to genotype these loci using haplotype tagging single-nucleotide polymorphisms (tSNPs) and a structural variant genotyping tool. These results provide a comprehensive view of VNTR variation and evolution in *MUC5AC* and *MUC5B* and outline a path forward for improved disease association studies.

Material and methods

Long-read sequence assembly and QC

Whole-genome assemblies from 104 HGSVC[9] ($n = 57$) and HPRC[10] ($n = 47$) samples were leveraged for *MUC5AC* and *MUC5B* variant discovery. The genome sequence data for both cohorts are consented for open access with no data use restrictions. These genomes include 49 Africans (AFR), 23 Admixed Americans (AMR), 14 East Asians (EAS), 10 Europeans (EUR), and 8 South Asians (SAS; Tables S1 and S2); these geographic population descriptors were defined previously by the HGSVC and HPRC. Sequencing for both cohorts was conducted using PacBio HiFi CCS. Average HPRC sequencing coverage was 42× (minimum = 31×) and average HPRC read N50 was 19.7 kbp (minimum = 13.5 kbp). Average HGSVC sequencing coverage was comparable at 40× (minimum = 25×) and average read N50 was 17.2 kbp (minimum = 10.0 kbp). The HPRC genome assembly was performed by Liao et al.[10] using trio-hifiasm[11] (maternal and paternal short reads used in haplotype phasing). We assembled 54 HGSVC samples using hifiasm version 0.16.1[11] (pseudo-haplotype resolved phasing). For the remaining three HGSVC samples with trio information (HG00514, HG03125, NA12878), we used paternal and maternal short reads with yak v.0.1 (https://github.com/lh3/yak) to create k-mer databases for contig phasing in the child's assembly with hifiasm v.0.15.1[11] (see Ebert et al.[9] for parental short-read information). The average HPRC haplotype assembly N50 was 40.8 Mbp (minimum = 17.4 Mbp) and average HGSVC haplotype assembly N50 was 55.2 Mbp (minimum = 14.1 Mbp). Regional assembly contiguity and reliability for the *MUC5AC/MUC5B* locus was assessed using the flagger pipeline[10] and Nucfreq, a method to detect potential misassemblies and collapses in phased haplotypes.[12] We also inspected for assembly misalignments using SafFire (https://github.com/mrvollger/SafFire).

We assessed 10 total NHP genome assemblies for chimpanzee ($n = 2$), bonobo ($n = 2$), gorilla ($n = 2$), Sumatran orangutan ($n = 2$), Bornean orangutan ($n = 1$), and Siamang gibbon ($n = 1$; Table S3). Specifically, these included PTR1 (Central chimpanzee, Clint), PPA1 (bonobo, Mhudiblu), GGO1 (Western gorilla, Kamilah), and PAB1 (Sumatran orangutan, Susie) and were assembled with hifiasm v.0.15.1.[13] All other NHP assemblies were generated as part of the Primate T2T (telomere-to-telomere) Consortium, and assemblies were downloaded from GenomeArk[14]; these include PTR2 (Central chimpanzee, AG18354), PPA2 (bonobo, PR00251), GGO2 (Western gorilla, Jim), PAB2 (Sumatran orangutan, AG06213), PPY1 (Bornean orangutan, AG05252), and SSY (Siamang gibbon, Jambi). The assemblies were constructed using both high-coverage PacBio HiFi CCS reads and ultra-long (UL) Oxford Nanopore Technologies (ONT) reads via the Verkko 2.0 assembler.[15] Information about assembly quality and validation can be found in Mao et al.[13] and Makova et al.[14] We inspected the *MUC5AC/MUC5B* regional assembly contiguity using SafFire in the same manner as the HGSVC assemblies.

Sequence extractions and phylogenetic analyses

HPRC, HGSVC, and NHP phased genome assemblies were aligned to CHM13[16] using minimap2 v.2.24[17] with CIGAR string inclusion, full-genome alignment divergence less than 10%, secondary alignments suppressed, and a minimal peak all-versus-all alignment score of 25,000. Coordinates for a specific locus in individual haplotype assemblies were identified using rustybam v.0.1.29 (https://github.com/mrvollger/rustybam), and sequences were extracted using seqtk v.1.3 (https://github.com/lh3/seqtk). Exon and intron boundaries were defined based on human GENCODE V35[18] annotations in CHM13[16] (GENCODE: MUC5AC-201, GENCODE: MUC5B-204). Intronic and intergenic sequences used to construct phylogenies were selected in a recombination-aware manner based on UCSC Genome Browser 1000 Genomes Project (1KG) linkage disequilibrium (LD) structure annotations.[19] A multiple sequence alignment (MSA) was conducted using MAFFT v.7.487[20] with global pairwise alignment and 100 iterations, followed by visual inspection of alignment quality using Jalview v.9.0.5.[21] Segments of the MSA determined to be misaligned were identified and eliminated manually. Maximum-likelihood tree calculations were performed using iqtree v.1.6.12[22] with automatic model selection and 1,000 bootstraps. All phylogenetic trees in figures were constructed using ggtree v.3.2.1[23] in R v.1.4.2 (https://www.R-project.org). Haplogroup coalescence times were estimated with iqtree2[24] based on estimated chimpanzee divergence (6.4 million years ago [mya]).[25]

Gene and protein domain/VNTR motif annotations

Computational protein prediction for all human and NHP haplotypes was conducted via the same alignment pipeline as phylogeny construction based on human exon annotations from CHM13.[16] We predicted translated exons using the ExPasy tool in EMBOSS v.6.6.0.[26] For computational protein predictions that were complete (i.e., complete open reading frame [ORF], no truncations), protein domain annotations were manually curated using cys domain and VNTR domain sequences previously annotated by Guo et al.[7] Protein groups (P1–P6) were defined for *MUC5AC* as containing more than one haplotype and variation in cys domain copy number, tandem repeat domain copy number, and/or repeat motif copy-number variation in homologous VNTR domains. Protein groups for *MUC5B* were similarly defined; however, the inclusion criteria of harboring more than one haplotype per group was dismissed due to protein sequence length variation in three singletons for *MUC5B* (P1, P4, P5). We characterized motif variation across individual VNTR domains for human *MUC5AC* and *MUC5B* based on previously published consensus motif

sizes (24bp/8 amino acids [aa] for *MUC5AC*,[27] 87bp/29aa for *MUC5B*[28]). Heatmaps of motif usage for all haplotypes of *MUC5AC* and *MUC5B* were constructed using a custom R script that included normalization on total VNTR sequence space (motif counts/total number of motifs) to account for length variability, normalization within motifs, and hierarchical clustering (unweighted pair group method of arithmetic mean [UPGMA] clustering[29]) of haplotypes and motifs for group visualization. Similarly, motif diagrams in linear sequence space were constructed using a custom R script that designated a unique color to each distinct motif and clustered unique alleles by row using UPGMA.

NHP allele alignments and intronic VNTR analysis

We generated all-versus-all alignments between the most common haplotypes of *MUC5AC* and *MUC5B* in humans and NHPs using minimap2[17] with the same parameters as phylogenetic analyses. Tiled alignment plots for each locus were constructed using SVbyEye v.0.99.0 (https://github.com/daewoooo/SVbyEye) in R v.4.3.1 with a bin size of 10,000 bp and custom percent identity breaks. VNTR sequences in intron 15 and ~3 kbp before the start codon of *MUC5AC* were curated using tandem repeats finder v.4.10[30] with the following parameters: match = 2, mismatch = 7, delta = 7, percent match (PM) = 80, percent indels (PI) = 10, minimum alignment score = 50, and max period size = 30. Detection of H3 k-mers for the intronic VNTR was conducted using STREME from the MEME suite of motif-based sequence analysis tools v.5.5.4.[31]

LD block structure and selection detection analyses

Illumina whole-genome sequencing (WGS) data from the most recent high-coverage (30×) 1KG release[19] were used to assess the LD structure of the *MUC5AC/MUC5B* locus. These data include open-access WGS from 2,600 unrelated individuals: 691 AFR, 526 EUR, 514 SAS, 515 EAS, and 354 American genomes. Population identifier acronyms are consistent with 1KG nomenclature and include the following: ACB = African Caribbean in Barbados, GWD = Gambian in Western Division, ESN = Esan in Nigeria, MSL = Mende in Sierra Leone, YRI = Yoruba in Nigeria, LWK = Luhya in Kenya, ASW = Americans of African Ancestry in SW USA, PUR = Puerto Rican in Puerto Rico, CLM = Colombian in Colombia, PEL = Peruvian in Peru, MXL = Mexican Ancestry in Los Angeles USA, GBR = British in England and Scotland, FIN = Finnish in Finland, IBS = Iberian in Spain, CEU = Utah residents (CEPH) with Northern/Western European ancestry, TSI = Toscani in Italy, PJL = Punjabi in Pakistan, BEB = Bengali in Bangladesh, STU = Sri Lankan in the UK, ITU = Indian Telugu in the UK, GIH = Gujarati from Houston TX USA, CHS = Southern Han Chinese, CDX = Chinese Dai in China, KHV = Vietnamese in Vietnam, CHB = Han Chinese in Beijing, China, JPT = Japanese in Japan. LDBlockShow v.1.40[32] was used to construct LD plots based on D′[33] for all SNPs in the *MUC5AC/MUC5B* region (GRCh38 coordinates, chr11:1,117,952–1,272,172). Autosome-wide LD block calculations were estimated with the PLINK v.1.9[34] blocks parameter, which estimates haplotype blocks based on definitions described by Gabriel et al.[35] (the region of chromosome 11 that harbors *MUC5AC* and *MUC5B* features a high recombination rate).[36] Calculations were limited to SNPs with a minor allele frequency greater than 5%, those with 75% or higher genotyping rate, and those in Hardy-Weinberg equilibrium. To assess whether the region of chromosome 11 containing *MUC5AC* and *MUC5B* shows signatures of selection, Tajima's D[37] analysis was conducted using the phased 1KG cohort of samples. Chromosomes were partitioned into 10 kbp bins with filtering for bins that contained at least 10 SNPs. Tajima's D statistics were computed for bins using PLINK v.1.9,[34] and regions harboring signatures of either positive or balancing selection were based on the 90th and 95th percentiles of values in the super population autosome-wide distributions (negative Tajima's D is suggestive of positive selection, positive Tajima's D is suggestive of balancing selection). Permutation testing with multiple-test correction was performed by randomly sampling 10,000 10 kbp nonoverlapping bins to produce a null distribution of Tajima's D values. This process was repeated 10,000× to produce a distribution of Tajima's D scores corresponding to the bottom and top 5th percentiles. p values per bins were calculated based on the empirical ranking of Tajima's D scores relative to this final distribution and were corrected for multiple testing (considering both number of bins and populations tested).

tSNPs and mapping of disease-relevant GWAS SNPs

To uncover SNPs in significant LD with VNTR haplogroups of *MUC5AC* and *MUC5B*, phylogenetic haplogroups from the HGSVC/HPRC genomes were encoded as biallelic SNPs. Calculation of squared correlations between these variants encoding haplogroup identity and all SNPs within 50 kbp of the loci were performed using PLINK v.1.9.[34] Genome-wide association study (GWAS) risk alleles for *MUC5AC* and the phenotypes of asthma/allergy and infection-induced pneumonia/meningitis were mined through the GWAS catalog.[38] Variants were included in subsequent LD analysis if they had a reported p value of 1×10^{-9} or smaller for the phenotype association, had the nucleotide annotation for the risk allele, and were unambiguously mapped to the HPRC/HGSVC genomes. The final set of variants included six SNPs from six GWASs (rs35225972,[39] rs11245962,[40] rs28415845,[41] rs11245979,[42] rs28737416,[43] and rs28729516[44]). Squared correlation values were calculated in the same manner as tSNP discovery.

Genotyping of *MUC5AC* haplogroups in 1KG populations using Locityper

MUC5AC/MUC5B genotyping was performed with Locityper v.0.10.9[45] and its dependencies SAMtools v.1.19,[46] jellyfish v.2.3.0,[47] and strobealign v.0.11.0.[48] Diploid genomes from the HGSVC/HPRC sample set were included as alleles in the reference panel if they were complete for the *MUC5AC/MUC5B* locus (no assembly breaks or alignment ambiguities), annotated for both haplogroups, and had accessible high-quality short reads through the 1KG dataset. The final set of genomes that constituted the reference panel included 99 genomes (i.e., 198 haplotypes) for *MUC5AC* and *MUC5B*.

The CHM13 reference genome[16] was used for all Locityper analyses, with gene coordinates set to chr11:1,227,366–1,274,380 and chr11:1,292,367–1,334,784 for *MUC5AC* and *MUC5B*, respectively. For leave-one-out analyses, the target sample for genotyping was excluded from database construction, and the highest alignment accuracy level was used. All other options for database construction, sequencing dataset preprocessing, and genotyping were set to default. Genotyping accuracy was determined based on edit distance (alignment differences) between the real and retrieved genotypes during leave-one-out analysis and compared to the closest "available" genotype (smallest edit distance between true genotype and all possible diploid combinations of alleles in the reference panel). Computation of edit distances between alleles in the leave-one-out concordance analysis was performed using the Locityper helper script "gt_dist.py."

MUC5AC and *MUC5B* phenome-wide association studies (PheWASs) in *All of Us*

Data from the *All of Us* Research Program[49] controlled tier database were analyzed for a phenome-wide association study (PheWAS) with the *MUC5B* promoter polymorphism rs35705950[50] and tSNPs for the major haplogroups of *MUC5AC* variants. All participants in the *All of Us* program provided electronic informed consent,[49] and the NIH *All of Us* IRB Operations Office determined this does not constitute research involving human subjects. As of January 2024, this cohort included ~245,400 individuals with short-read WGS data, of which ~185,000 were unrelated, annotated for age/sex, and had paired electronic health record (EHR) data (reported as International Classification of Diseases [ICD] codes). These individuals were categorized previously by *All of Us* for genetic ancestry using principal component analysis. We surveyed samples from African, European, East Asian, Admixed American, and Middle Eastern ancestries for *MUC5B* rs35705950 and tSNPs in high LD with *MUC5AC* haplogroups H1 (rs2075842, rs1132433, rs1132434, rs28652890, rs879136008), H2 (rs1015856541, rs28519516, rs28558973, rs28368633), and H3 (rs36154966, rs1004828576, rs940158763, rs36151150, rs36132281, rs35779873). We only included samples with genome quality scores $\geq$20 at individual loci; therefore, the final sample sets included ~32,500 AFR, ~3,200 EAS, ~2,000 SAS, ~98,600 EUR, ~28,200 ADM, and ~650 individuals of Middle Eastern ancestry, totaling ~165,150 individuals (exact number of individuals varied between locus associations in respective populations; Tables S7–S21). We included both ICD-9 and ICD-10 phenotype codes from patient EHRs and samples with male/female self-reported biological sex aged 20 years or older.

PheWAS analysis was performed using the R package PheWAS as outlined in Bick et al.[49] The package translated ICD-10 codes to ICD-9 and calculated case and control genotype distributions, allelic *p* value, and allelic odds ratio (OR) for each condition. A minimum count of two related codes was used to determine whether a phenotype was sufficiently represented in the health data for association. Sex at birth, age at sample collection, and principal component analyses 1–3 were used as covariates. The aggregate.fun function was used to correct for duplicates in the EHR. Nominal p was set to $<$2.7E−5 (p adjusted $<$ 0.05 after Bonferroni correction) for phenotype associations with rs35705950 and *MUC5AC* tSNP alleles in the dataset.

Results

MUC5AC/MUC5B assembly and QC

We performed targeted assessment of a ~160 kbp region of chromosome 11 spanning *MUC5AC* and *MUC5B* from 104 human genomes, including 47 genomes from the HPRC and 57 from the HGSVC where long-read sequencing data had recently been generated and made publicly available.[9,10] We generated phased genome assemblies from HGSVC samples using the same computational pipeline used for the generation of HPRC assemblies (Material and methods) from HiFi PacBio sequencing data. The combined sample set includes 49 AFR, 23 ADM, 14 EAS, 10 EUR, and 8 SAS (Material and methods, Tables S1 and S2). Next, we applied the flagger[10] and Nucfreq[12] computational pipelines to detect collapses or misassemblies across the 160 kbp target region. Of the 208 total human haplotypes, 206 (99%) were correctly assembled without gaps, breaks, or misjoins in the *MUC5AC/MUC5B* region. Two haplotypes (one each) from samples HG01114 and HG02509 were fragmented and excluded from further analyses.

For comparative evolutionary purposes, we analyzed 10 individuals from six NHP species for which HiFi sequencing data have recently been generated[13,14] (Material and methods, Table S3). In the NHP genomes, all *MUC5AC* loci passed quality control (QC) with no ambiguous alignments to CHM13; in contrast, one gorilla haplotype (Kamila h2) and both haplotypes of a Sumatran orangutan (Susie h1 and h2) failed *MUC5B* QC and were excluded from further analyses.

Human *MUC5AC* genetic and protein diversity

To understand human genetic diversity in *MUC5AC*, we first constructed a phylogeny centered around the gene model. We extracted 26.5 kbp of noncoding sequence flanking *MUC5AC* exons for the 206 human haplotypes and generated a maximum likelihood phylogenetic tree using chimpanzee as an outgroup. Human alleles were grouped into three distinct haplogroups or clades (Figure 1A), namely H1 (n = 103), H2 (n = 78), and H3 (n = 25). H1 is the most phylogenetically distinct (100% bootstrap support), is reduced in frequency among AFR genomes ($p = 4 \times 10^{-3}$ comparing H1 to H2/H3 frequencies via chi-square, Figure 1B), and is estimated to have arisen most recently. We estimate an H1 coalescent of ~120,000 years ago when compared to H2 or H3 (~330,000 years ago).

Next, we predicted a protein model associated with each human haplotype (Material and methods). We identified 16 distinct MUC5AC protein variants with extensive length variation (Figure 1C). The three most common protein variants, 5,654 aa/96 haplotypes, 5,742 aa/67 haplotypes, and 6,325 aa/15 haplotypes (Figure 1C), project onto the phylogenetic haplogroup designations H1, H2, and H3, respectively. There is, however, additional variation not immediately apparent from the phylogeny that is uncovered by detailed protein sequence curation. Guo et al.[7] classified protein variants into three groups (P2, P3, and P6) based on MUC5AC domain annotations. We extend this classification by identifying three additional protein variant groups (P1, P4, and P5) based on VNTR domain, cys domain, and VNTR motif copy-number variation.

Most MUC5AC protein variants harbor four distinct tandem repeat domains (P1–3, P5); however, two groups (P4, P6) harbor an additional central domain with 11 copies of the 8-mer repeat motif. P5/6 variants harbor additional type 2 and type 3 cys domains, while P1 variants harbor a novel deletion of these domains. VNTR motif copy-number variation is also extensive in the first and last domains across variant groups.

We characterized the composition of the MUC5AC VNTR 8-mer repeat because the density of glycosylated serines and threonines is critical for mucin barrier function. We find that the absolute count of serine and threonine

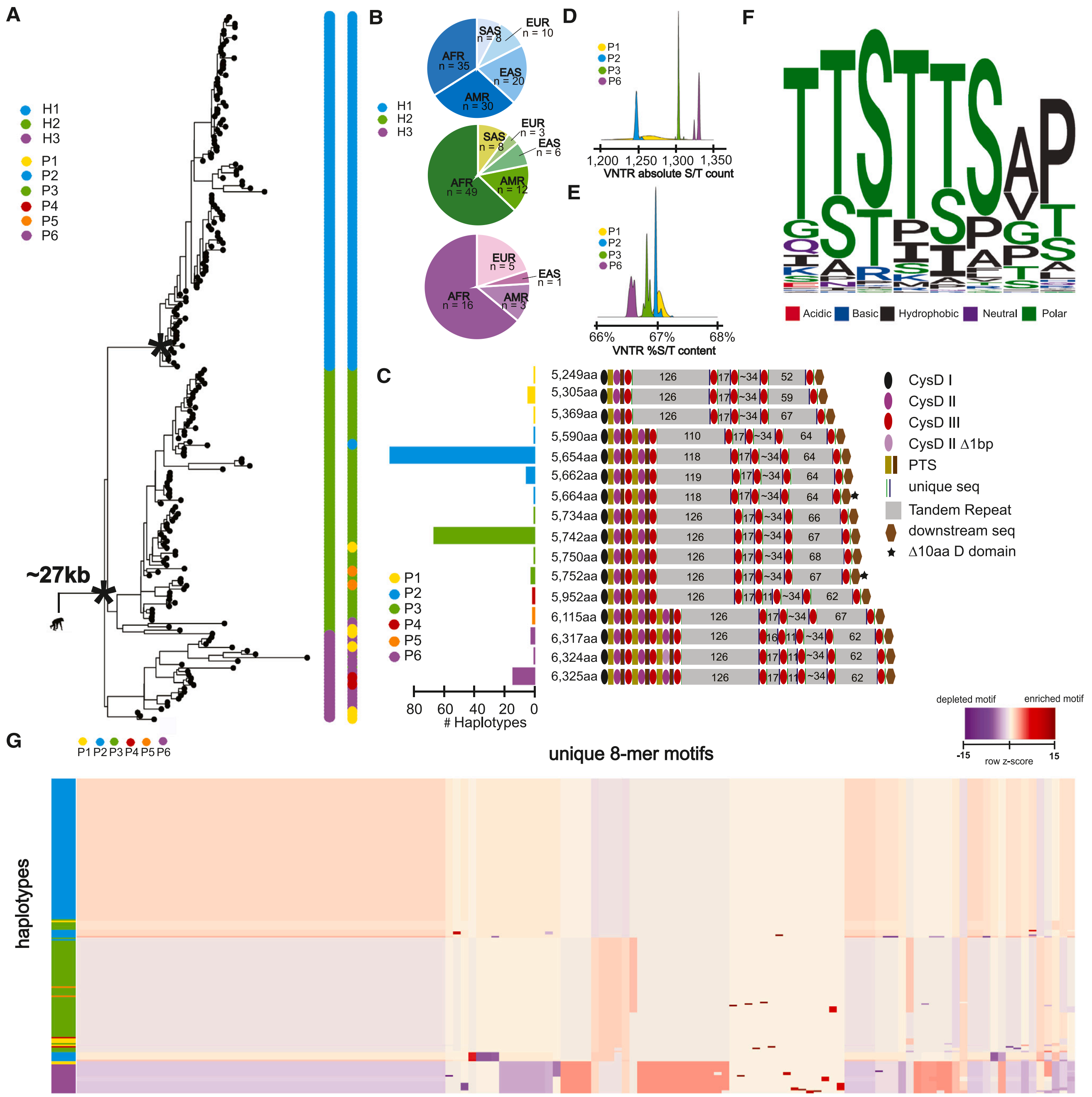

Figure 1. The genetic architecture of *MUC5AC* in 206 human haplotypes
(A) Recombination-aware phylogenetic analysis of ~27 kbp neutral sequence (5.592 kbp from introns 31–48 and 21 kbp from 3′ flanking sequence) from 206 human haplotypes of *MUC5AC* with two chimpanzee haplotypes as outgroup. (*) = central node with 100% bootstrap support. H1–H3 correspond to three major haplogroups; P1–P6 correspond to protein groups (consistent with C).
(B) Frequency of population-specific haplotypes found in the three common phylogenetic haplogroups of *MUC5AC*. H1–H3 correspond to the three major haplogroups.
(C) Protein predictions for haplotypes of *MUC5AC*. Diagrams represent protein domains with the large central exon of *MUC5AC*, modeled after Guo et al.[7] Colors correspond to protein groups visualized in (A). CysD corresponds to cys domains and PTS corresponds to proline-, serine-, and threonine-rich domains.
(D) Distributions of absolute serine and threonine (S/T) count across VNTR domains within the four most common protein groups of MUC5AC.
(E) Distributions of percent S/T content within VNTR domains for the four most common protein groups of MUC5AC.
(F) Logo plot of the 130 8-mer amino acid motif variants used in MUC5AC VNTR domains. Colors correspond to biochemical groupings of amino acids.
(G) Heatmap of 8-mer motif utilization across 206 protein variants of human MUC5AC, colored vertically by protein group identities. Heatmap constructed with normalization within motifs (columns) and hierarchical clustering of haplotypes (rows) and motifs (columns). See Figure S2 for an extended version that includes the matched motifs (columns).

residues across the VNTR domains is positively correlated with protein length (Figure 1D); however, when normalized for the total length of the VNTR, the two shortest protein variant groups (P1 and P2) harbor the highest concentration of serines and threonines (Figure 1E). There are a remarkable 211 unique 24-mers (nucleotides) and 130 unique protein 8-mer motifs (aa) diversifying the degenerate VNTR domains; motif changes, however, are constrained, with most harboring the pattern of TTSTTS in the first six aa (Figures 1F and S1). The preferential use of threonines is likely a consequence of the higher propensity for threonines to harbor O-glycans,[51] thereby facilitating MUC5AC barrier functionality. Furthermore, the high incidence of prolines likely contributes to the glycosylation potential of nearby serines/threonines by exposing these residues in a β-turn conformation.[52]

Of the 130 unique protein 8-mers for MUC5AC, only nine are unique to a single haplotype, indicating that most motif variation is shared between protein isoforms. There are distinctive modules of motifs that cluster together in frequency of usage for protein groups 2, 3, and 6 (Figures 1G and S2). Most motif variation is due to single nonsynonymous aa changes between haplotypes; however, there are instances where entire motifs have been gained or lost. Overall, there is extensive cys domain copy number, VNTR copy number, and VNTR motif usage variation in the large central exon of *MUC5AC* across human haplotypes.

Human *MUC5B* genetic and protein diversity

Similarly, we analyzed the *MUC5B* locus and observed far less genetic and protein variability compared to *MUC5AC*. A maximum likelihood phylogenetic tree (24.6 kbp intronic sequence using chimpanzee as an outgroup) distinguishes two distinct human haplotypes with 100% bootstrap support (Figure 2A). The most common haplogroup H2 was identified in 82% (169/206) of assembled haplotypes and is estimated to have emerged ~770,000 years ago. The less abundant H1 (18%) variants predictably arose more recently (~407,000 years ago). While H2 is found across all continental populations, H1 shows a notable reduction in East Asians (Figure 2B). At the protein level, we predict a complete ORF for 92% (190/206) of haplotypes and a premature stop codon for ~8% (16/206, Figure 2C). We hypothesize that these haplotypes harbor assembly artifacts due to the homopolymer runs within the *MUC5B* VNTR. To test this, we reassembled eight of the samples where both ONT and HiFi sequence data were available[15] and recovered the ORF for three. These haplotypes harbored predicted protein lengths consistent with P3 (5,762 aa).

Among the 190 haplotypes with complete ORFs, 87% predict proteins with the canonical MUC5B length of 5,762 aa (P3). The second most abundant, P2, differs in length by one aa (5,761 aa) and represents 9% of protein isoforms. These findings support the long-standing belief that *MUC5B* is less variable than *MUC5AC*.[54] Our deeper survey, however, suggests that the locus is not invariant. We identify seven haplotypes (3.7%, 7/190 complete proteins) where the protein is predicted to have elongated (6,291–7,019 aa, P4–P6) due to expansion of the VNTR domains. Five of these variants harbor seven VNTR domains with an excess of ~800 aa of tandem repeat sequence and two additional cys domains. Unlike *MUC5AC*, there is no variation in cys domain copy number preceding the first tandem repeat domain in *MUC5B*. All elongated variants were found exclusively in individuals of African descent; therefore, much like *MUC5AC*, the ancestral state of this locus may have been longer.

The novel VNTR domains associated with P4–P6 are most like TR3 and TR4 in repeat copy number and motif composition (Figure S3), suggesting that the acquisition of new tandem repeat domains has been accomplished via duplication of the central domains in *MUC5B*, rather than from the first and last domains. While the largest MUC5B protein isoform (P6) has increased in size due to VNTR expansion, it is interesting that serine and threonine abundance is comparable to that of the canonical forms (P1–P4) (Figures 2D and 2E). Like MUC5AC, threonine is favored across the irregular MUC5B repeat motif (Figure 2F). Even though there are fewer distinct MUC5B protein variants, there are 191 unique 29-mers used across the haplotypes (Figures 2G and S4). Unlike *MUC5AC*, there appear to be no gains or losses of whole motifs, and the frequency of motif usage is largely conserved across the haplotypes (Figure 2B).

NHP variation in *MUC5AC* and *MUC5B*

We reconstructed the evolutionary histories of *MUC5AC* and *MUC5B* by identifying orthologous loci from NHP genomes,[13,14] including chimpanzee ($n = 2$), bonobo ($n = 2$), gorilla ($n = 2$), orangutan ($n = 3$, Sumatran and Bornean species), and Siamang gibbon (Figure 3; Table S3). All NHP haplotypes ($n = 22$) predicted a complete ORF at the *MUC5AC* locus, consistent with the human exon structure. Chimpanzee and bonobo alleles display variation in the number of cys domains preceding the first tandem repeat domain (Figure 3A). The Asian apes, orangutan, and gibbon carry the longest predicted proteins, with the most common protein variant in orangutan approximately 1,500 aa longer than the human H3 variant. All NHP variants were longer than the two most common human variants (H1 and H2), ranging in size from 6,243–7,887 aa, due solely to exon 31 length variation (Figure 3B). This suggests there has been a reduction of the VNTR length in humans (Figures 3B and 3C).

Additionally, we characterized two noncoding VNTRs associated with the *MUC5AC* locus—an 8-mer VNTR in intron 15 of *MUC5AC* (Figures 3C and S5, and Note S1) and an 8-mer VNTR approximately 1–3 kbp in size mapping upstream of the *MUC5AC* start codon (Figure S6). Based on ENCODE H3K27 mapping data,[18] the latter region corresponds to a potential enhancer. Diminished

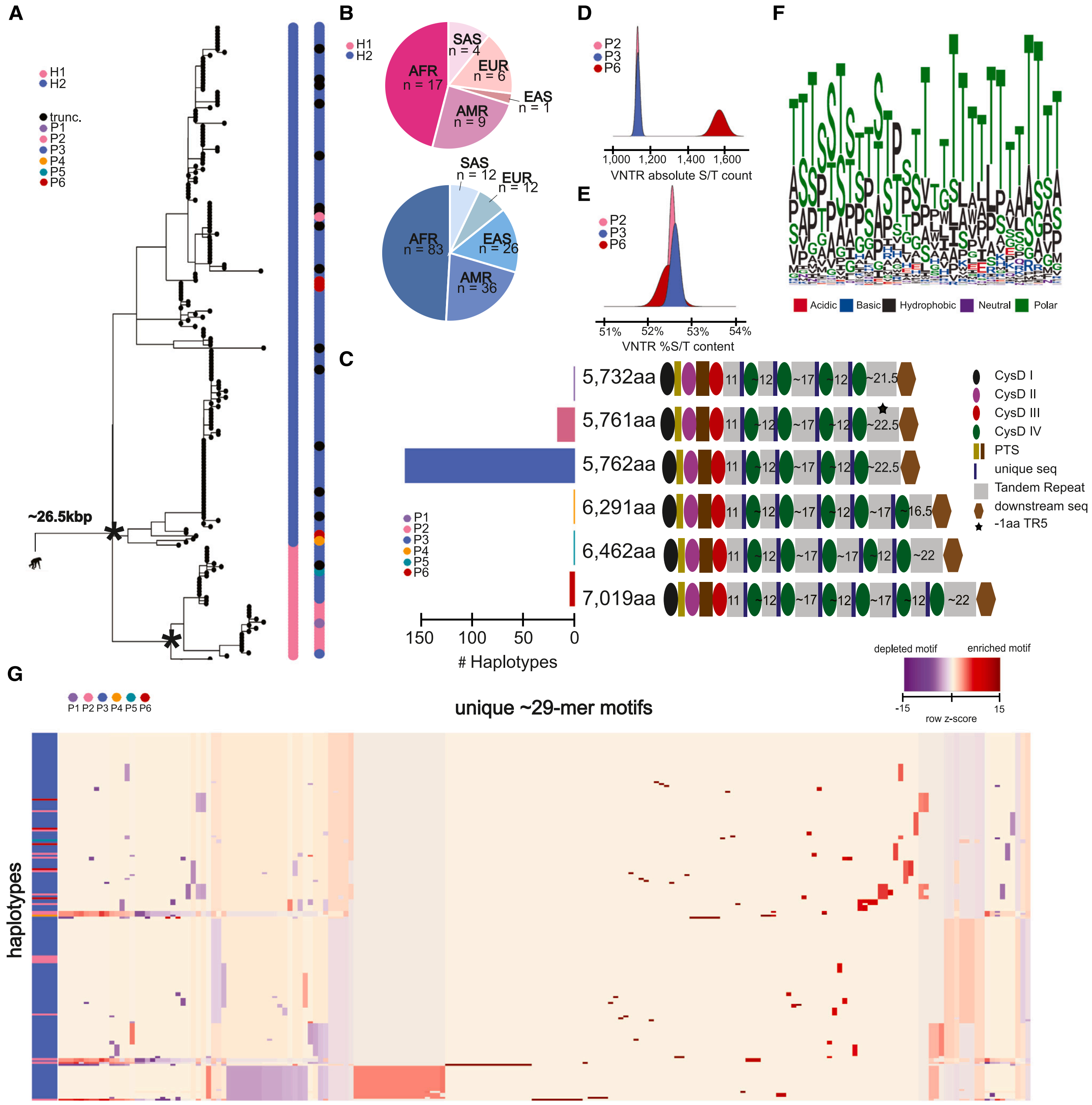

Figure 2. The genetic architecture of *MUC5B* in 206 human haplotypes

(A) Recombination-aware phylogenetic analysis of ∼26.5 kbp neutral sequence (introns 16–48) from 206 human haplotypes of *MUC5B* with two chimpanzee haplotypes as outgroup. (*) = central node with 100% bootstrap support. H1 and H2 correspond to two major haplogroups; P1–P6 correspond to protein groups (consistent with C); trunc. corresponds to haplotypes with truncated protein predictions.

(B) Frequency of population-specific haplotypes found in the two common phylogenetic haplogroups of *MUC5B*.

(C) Protein predictions for 206 human haplotypes of *MUC5B*. Diagrams represent protein domains with the large central exon of *MUC5B*, modeled after those in Ridley et al.[53] Colors correspond to protein groups visualized in (A). CysD corresponds to cys domains and PTS corresponds to proline-, serine-, and threonine-rich domains.

(D) Distributions of absolute serine and threonine (S/T) count across VNTR domains for the three most common protein groups of MUC5B.

(E) Distributions of percent S/T content within VNTR domains for the three most common protein groups of MUC5B.

(F.) Logo plot of the complete 29-mer amino acid motif variants used in MUC5B VNTR domains across 206 human haplotypes. Colors correspond to biochemical groupings of amino acids.

(G) Heatmap of 190–29-mer motif utilization across protein variants of human MUC5B, colored vertically by protein group identities. Heatmap constructed through normalization for total VNTR sequence length, normalization within each motif (columns), and hierarchical clustering of haplotypes (rows) and motifs (columns). See Figure S4 for an extended version that includes the matched motifs (columns).

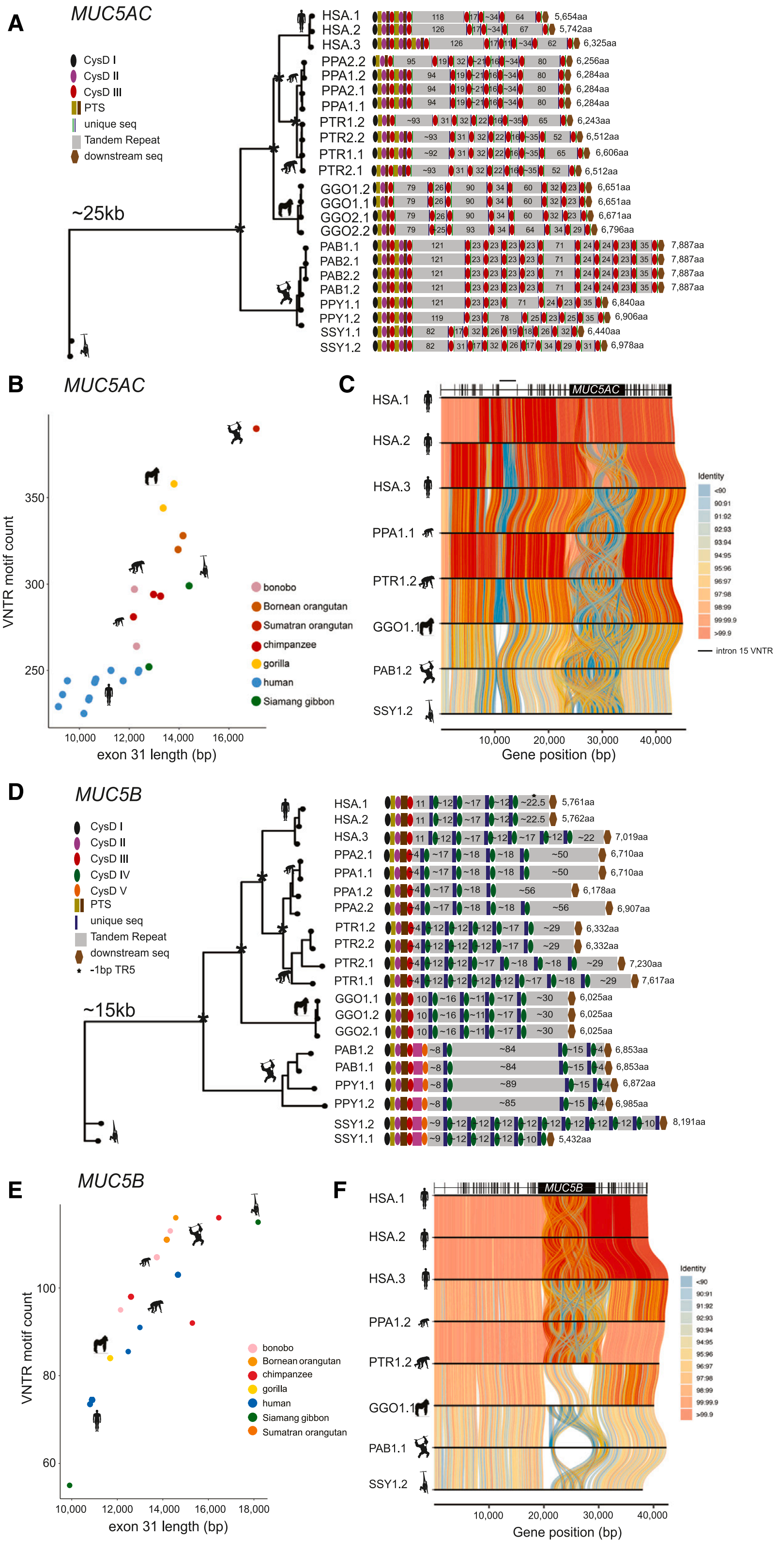

Figure 3. The genetic architecture of *MUC5AC* and *MUC5B* in the nonhuman ape lineages

(A) Phylogenetic analysis of ∼25 kbp from at minimum two haplotypes per ape lineage for *MUC5AC* and subsequent protein predictions based on human exon boundary alignments. (*) = central node distinguishing species branches with bootstrap support. Diagrams represent protein domains within the large central exon. HSA denotes human haplotypes.

(B) Scatterplot of total *MUC5AC* exon 31 length (in base pairs) and total VNTR motif count across all VNTR domains in human and NHPs.

(C) Tiled alignments between representative haplotypes of each ape species (most common or most structurally unique haplotype per species) for *MUC5AC*. *MUC5AC* intron/exon boundaries are distinguished by the gene model at the top of the visualization.

(D) Phylogenetic analysis of ∼15 kbp from at minimum two haplotypes per NHP lineage and subsequent protein predictions for *MUC5B* haplotypes based on human exon boundary liftover. (*) = central node distinguishing species branches with 100% bootstrap support. Diagrams represent protein domains with the large central exon.

(E) Scatterplot of total *MUC5B* exon 31 length (in base pairs) and total VNTR motif count across all VNTR domains in human and NHPs.

(F) Tiled alignments between representative haplotypes of each NHP species (most common or most structurally unique haplotype per species) for *MUC5B*. *MUC5B* intron/exon boundaries distinguished by gene model at top of visualization.

copy number of the enhancer VNTR is associated with decreased *MUC5AC* expression[55] and susceptibility to severe gastric cancer.[56] We find complete enrichment of shorter variants (less than 1,500 bp in length) in East Asian H1 haplotypes and an excess of long variants (greater than 2,000 bp) in African haplotypes ($X^2 = 87.4$, $p < 0.001$), suggesting a founder effect or selection among East Asians consistent with their eastward expansion that could result in population-specific differential expression of H1. Additionally, all NHP haplotypes feature lengths of 881–1,649 bp for this enhancer VNTR (shortest in orangutan and longest in chimpanzee).

Despite enhanced conservation in humans, there is extensive length variation among the protein-coding MUC5B variants among great apes. Only orangutan and gibbon haplotypes harbor an additional cys domain that is distinctive from the other three cys domain types (Figure 3D), which we classify as a type V domain. Like *MUC5AC*, orangutans carry the largest *MUC5B* VNTR domains (84–89 copies of the 29-mer). Excluding one haplotype from the Siamang gibbon, human alleles of *MUC5B* generally harbor shorter central exons with fewer VNTR total motifs compared to the NHP haplotypes (Figure 3E) and little structural variation outside of the central exon (Figure 3F).

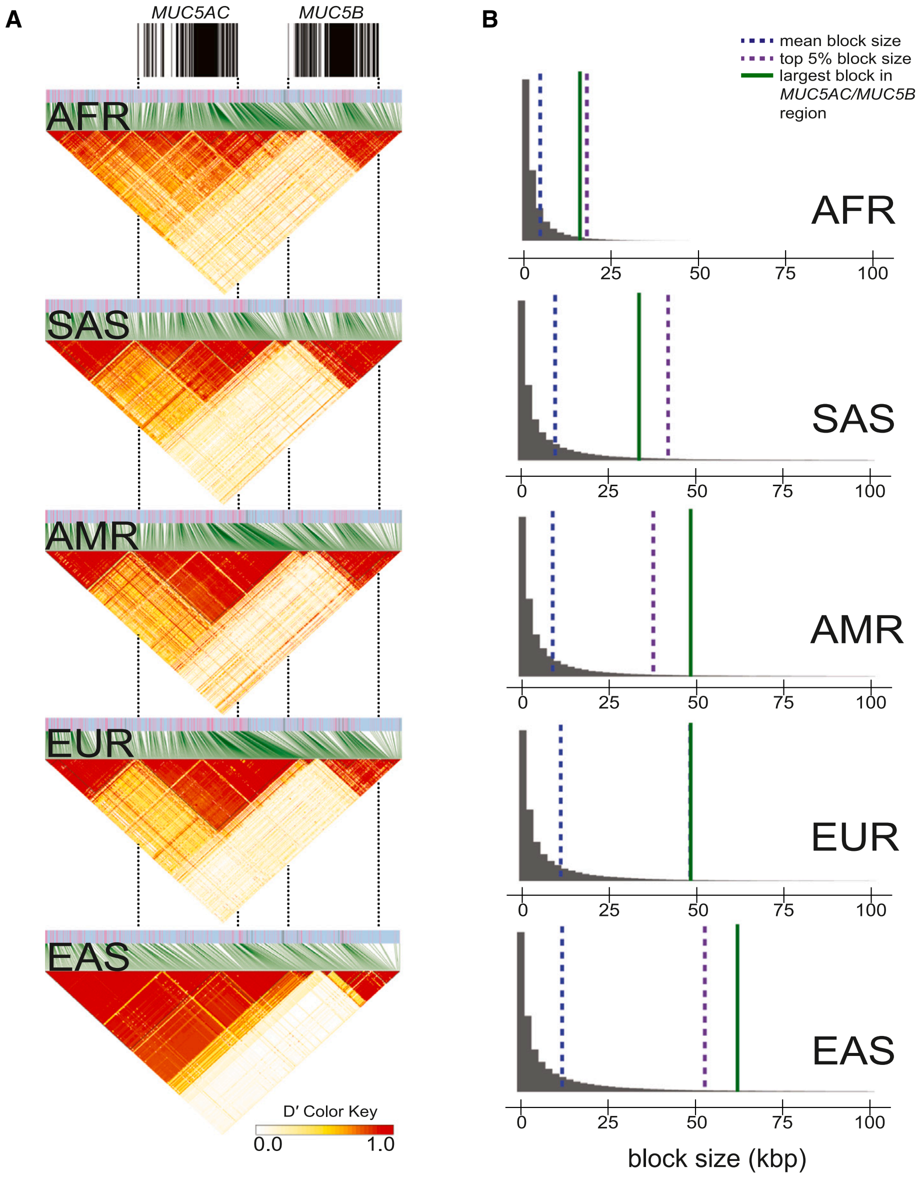

Figure 4. Linkage disequilibrium (LD) analysis of the *MUC5AC/MUC5B* locus for African, American, European, East Asian, and South Asian genomes from the phased, short-read 1000 Genomes Project (1KG) cohort

(A) LD plots for the *MUC5AC/MUC5B* locus based on D′, with increasing red intensity indicative of higher LD between SNPs. Gene models corresponding to *MUC5AC* and *MUC5B* indicated by black annotations at top.

(B) Autosome-wide LD block size distributions for each major population. Blocks above 100 kbp visually excluded as outliers (included in distribution analyses within populations).

MUC5AC LD block structure and potential positive selection in East Asian populations

We next investigated LD patterns among different human continental groups using D′ at the *MUC5AC/MUC5B* locus. A predominant single LD block corresponded to most of the MUC5AC protein-coding genes (Figure 4A) in the non-African populations. We tested by simulation (Figure 4B) whether LD block sizes were significantly larger than the genome-wide distributions because extended LD is a signature of positive selection. When compared to population-specific distributions of LD block sizes in the 1KG dataset,[19] *MUC5AC* blocks are large (top 5% distribution) in EAS ($n = 585$) and Americans ($n = 490$) relative to AFR ($n = 893$), EUR ($n = 633$), and SAS ($n = 601$, Figure 4B).

To further test for positive selection, we calculated Tajima's D[37] for 10 kbp segments spanning *MUC5AC* and *MUC5B* in the 1KG sample set. We find a significant excess of rare variants in four bins within *MUC5B* for Africans and one bin for East Asians, consistent with positive selection (Table 1). Repeating the analysis for *MUC5AC*, only one population group (East Asians, Table 2) shows a significantly negative Tajima's D value corresponding to the 10 kbp segment preceding the VNTR. East Asians are the only population with both an excess of rare variants and

Table 1. Tajima's D statistic for *MUC5B* in the 1KG

			GRCh38 chromosome 11 bin					
Population	Gene	–	1,220,000	1,230,000	1,240,000[a]	1,250,000[a]	1,260,000	1,270,000
AFR	*MUC5B*	Tajima's D	−1.09	−1.47	−1.84[c,d]	−1.87[c,d]	−1.94[c,d]	−2.10[c,d]
		# SNPs	180	162	444	279	209	248
EUR	*MUC5B*	Tajima's D	−0.44	−0.42	−1.49	−1.39	−1.56	−1.60
		# SNPs	122	81	356	191	73	103
SAS	*MUC5B*	Tajima's D	−0.55	−0.96	−1.76[b]	−1.77[b]	−1.59	−1.65
		# SNPs	140	100	328	188	85	102
EAS	*MUC5B*	Tajima's D	−0.25	−0.49	−1.58	−1.69[b]	−2.13[c,d]	−1.91[b]
		# SNPs	109	62	189	109	79	83
AMR	*MUC5B*	Tajima's D	−0.46	−1.22	−1.84	−1.87[b]	−1.95[b]	−1.90[b]
		# SNPs	130	110	353	201	108	122

Bin sizes of 10 kbp were used to compare values to the autosome-wide distribution per population in the 1KG cohort.[19]
[a]Corresponds to bin containing VNTR sequence.
[b]Bottom 10% of autosome-wide Tajima's D values.
[c]Bottom 5% of autosome-wide Tajima's D values.
[d]Significant at $\alpha = 0.05$ after permutation testing.

an abnormally large block of LD for *MUC5AC*, thereby providing more compelling evidence of positive selection.

tSNP discovery and short-read genotyping using Locityper

We next searched for tSNPs in high LD with VNTR haplogroups for the imputation of structural variants in short-read WGS datasets. To discover tSNPs, we encoded H1, H2, and H3 as biallelic variants and tested for correlation (r^2) with all SNPs within 10 kbp of the *MUC5AC* start and stop sites (VNTR excluded). At a threshold of $r^2 > 0.85$, we discovered 35 tSNPs for H1 (max $r^2 = 0.92$), 5 tSNPs for H2 (max $r^2 = 0.89$), and 52 tSNPs for H3 (max $r^2 = 1$, Table S4). tSNPs for H3 are in low LD with H1/H2 and make excellent imputation candidates for this group of variants (average H1/H2 $r^2 = 0.10$). We found one tSNP distinguishing H1 and H2 of *MUC5B* that met our stringent criteria (in GRCh38, chr11:1,244,757; H1 $r^2 = 0.0026$ vs. H2 $r^2 = 1$).

Next, we applied Locityper—a tool designed to genotype complex, multi-allelic loci like *MUC5AC/MUC5B*—to WGS datasets.[45] Given a collection of high-quality reference alleles, Locityper predicts the best pair of alleles for an unknown sample by examining read alignments and read-depth profiles across all allele pairs. Locityper has a short runtime that allows thousands of genomes to be rapidly characterized. We tested the accuracy of Locityper in predicting haplogroup identities for *MUC5AC* and *MUC5B* in the HPRC/HGSVC genomes by performing leave-one-out experiments. For *MUC5AC*, we estimated a genotyping accuracy of 95% for full diploid genotyping (both haplogroups correct) and 97.5% concordance for partial genotyping (one haplogroup correct; Material and methods, Figure 5A; Table S5). For *MUC5B*, genotyping showed 100% accuracy in predicting the correct haplotype based on leave-one-out experiments (Figure S7; Table S5). Predictably, Locityper was less accurate in identifying protein isoforms due to homoplasy. For example, 91% and 81% of samples were correctly assigned to protein subgroups for *MUC5AC* and *MUC5B*, respectively (Table S5). A larger sampling of reference haplotypes will improve future genotyping with this tool.

Next, we genotyped all 2,600 unrelated genomes from the 1KG with high-coverage short-read Illumina WGS.[19] We compared concordances for two high-confidence tSNPs with Locityper predictions for *MUC5AC* (H1 vs. H2/H3 tSNP: rs28542750, H3 vs. H1/H2 tSNP: rs769768817; Table S4). We found high concordance between the two methods, with 91% ($n = 2{,}359$) of the genomes yielding complete concordance with both haplotypes. For the remaining ~9% ($n = 241/2{,}600$), most were discordant for only one of the two haplotypes (92%, $n = 222/241$) and differed for classification of H1 versus H2 alleles (75%, $n = 166/222$).

We leveraged the Locityper set of haplogroup predictions to assess population patterns of *MUC5AC* variation. We find that H2 is enriched in AFR genomes (47% of all AFR haplotypes), while H3 is found predominantly among Africans and Europeans (18% and 21%, respectively; Figure 5B). In sharp contrast, H3 is virtually absent among East Asians (0.37%); we identify only four haplotypes found exclusively among Vietnamese. It is interesting that among South Asians, H3 once again rises to common allele frequency (~5%).

Using Locityper genotypes, we tested again for signatures of positive selection with Tajima's D (Figure 5C; Table 3). Our results suggest signatures of positive selection for H1 in EAS and SAS. We find that H2 in AFR yields a significantly negative Tajima's D value in the bin of *MUC5AC* preceding the tandem repeats, unlike the other

Table 2. Tajima's D statistic for *MUC5AC* in the 1KG

			GRCh38 chromosome 11 bin						
Population	Gene	–	1,150,000	1,160,000	1,170,000	1,180,000[a]	1,190,000[a]	1,200,000	1,210,000
AFR	*MUC5AC*	Tajima's D	−1.06	−1.57	−1.37	−1.25	−0.98	−0.57	−1.23
		# SNPs	213	268	428	259	241	226	166
EUR	*MUC5AC*	Tajima's D	−0.48	−1.37	−0.85	−0.54	0.17	0.52	−0.66
		# SNPs	116	193	304	186	148	110	82
SAS	*MUC5AC*	Tajima's D	−0.70	−1.52	−1.63	−1.46	−0.71	−0.28	−0.87
		# SNPs	134	191	312	194	154	132	92
EAS	*MUC5AC*	Tajima's D	−0.62	−1.74[b]	−2.04[c,d]	−1.70[b]	−0.94	−0.31	−1.28
		# SNPs	105	173	292	152	127	100	85
AMR	*MUC5AC*	Tajima's D	−0.79	−1.54	−1.39	−1.26	−0.93	−0.90	−1.29
		# SNPs	140	196	300	192	183	170	109

Bin sizes of 10 kbp were used to compare values to the autosome-wide distribution per population in the 1KG cohort.[19]
[a]Corresponds to bin containing VNTR sequence.
[b]Bottom 10% of autosome-wide Tajima's D values.
[c]Bottom 5% of autosome-wide Tajima's D values.
[d]Significant at $\alpha = 0.05$ after permutation testing.

super populations examined. In contrast, we find significantly positive Tajima's D values for *MUC5AC* H3 in EUR, ADM, and SAS. We tested for departure from Hardy-Weinberg equilibrium and found a significant depletion of homozygotes in Africans and Europeans (chi-squared test, AFR: $p = 0.0368$, EUR: $p = 0.030$), consistent with the action of balancing selection. These combined selection signatures in Europeans suggest there is an immunological advantage to shorter haplotypes of *MUC5AC* and heterozygote advantage for the longer alleles (H3).

MUC5AC haplogroups in LD with GWAS risk SNPs and expression quantitative trait loci (eQTLs)

Because the tSNPs we uncovered are unlikely to be genotyped in previous GWASs, we assessed the LD of *MUC5AC* haplogroups with risk and protective alleles for asthma/allergy phenotypes and infection-induced pneumonia/meningitis. The risk alleles for three SNPs associated with asthma/allergy (rs35225972,[39] rs11245962,[40] and rs28415845[41]; EUR cohorts) are in moderate LD with H1 variants of *MUC5AC* (Figure 5D). Conversely, the protective alleles for two SNPs associated with infection-induced pneumonia/meningitis (rs11245979,[42] rs28729516[44]; EUR cohorts) are in higher LD with H1, with rs28729516 functioning as a tSNP for this haplogroup. We also examined SNP-associated expression quantitative trait loci (eQTLs) for *MUC5AC* identified in the upper airways of African American and Hispanic children.[57] These eQTLs were parsed into two independent groups related to increased (group A) and decreased (group B) *MUC5AC* expression. We found that group A eQTLs (increased *MUC5AC* expression/decreased lung function) have an average r^2 of 0.79 for the risk variant and H1 *MUC5AC* alleles, whereas group B eQTLs (decreased *MUC5AC* expression) have an average r^2 of 0.82 for the protective variant and H3 (Table S6). These findings suggest that differences in VNTR structure are likely important considerations for differential *MUC5AC* expression.

MUC5AC and *MUC5B* PheWAS in *All of Us*

To identify phenotypes associated with *MUC5AC* and *MUC5B* variation, we performed a PheWAS using data from *All of Us*[49] ($n = \sim$165,150). We first tested for a known disease association with the *MUC5B* regulatory polymorphism (rs35705950) and interstitial lung diseases.[50] We find significant associations after Bonferroni correction for rs35705950 in all samples (including age, sex, and PCs1–3 as covariates) with the ICD codes for alveolar and parietoalveolar pneumonopathy (p = 6.89E−44, OR = 2.05), idiopathic fibrosing alveolitis (p = 2.14E−36, OR = 2.85), postinflammatory pulmonary fibrosis (p = 2.62E−34, OR = 1.82), extrinsic allergic alveolitis (p = 3.96E−08, OR = 2.38), bronchiectasis (p = 9.77E−7, OR = 1.32), and pulmonary congestion and hypostasis (p = 2.25E−05, OR = 1.30; Figure S8; Table S7). Two or more of these phenotypes were associated with rs35705950 in Admixed Americans and Europeans when tested alone (Tables S8 and S9).

We find no correlated phenotypes that survive multiple testing correction for *MUC5AC* H1, H2, and H3 tSNPs (Material and methods). It is interesting to note, however, that H3 tSNPs approached significance for protection against degeneration of the macula and the posterior pole of the retina (p = 1.76E−4–9.49E−4, OR = 0.91; Table S19). We repeated the analysis separately for heterozygotes and homozygotes at rs36151150 (*MUC5AC* H3 tSNP) and find increased significance for the protective phenotype among heterozygotes, despite a reduction in alleles upon removal of homozygotes (heterozygous: p = 2.41E−4, OR = 0.89; homozygous: p = 0.145, OR = 0.94; Tables S20 and S21).

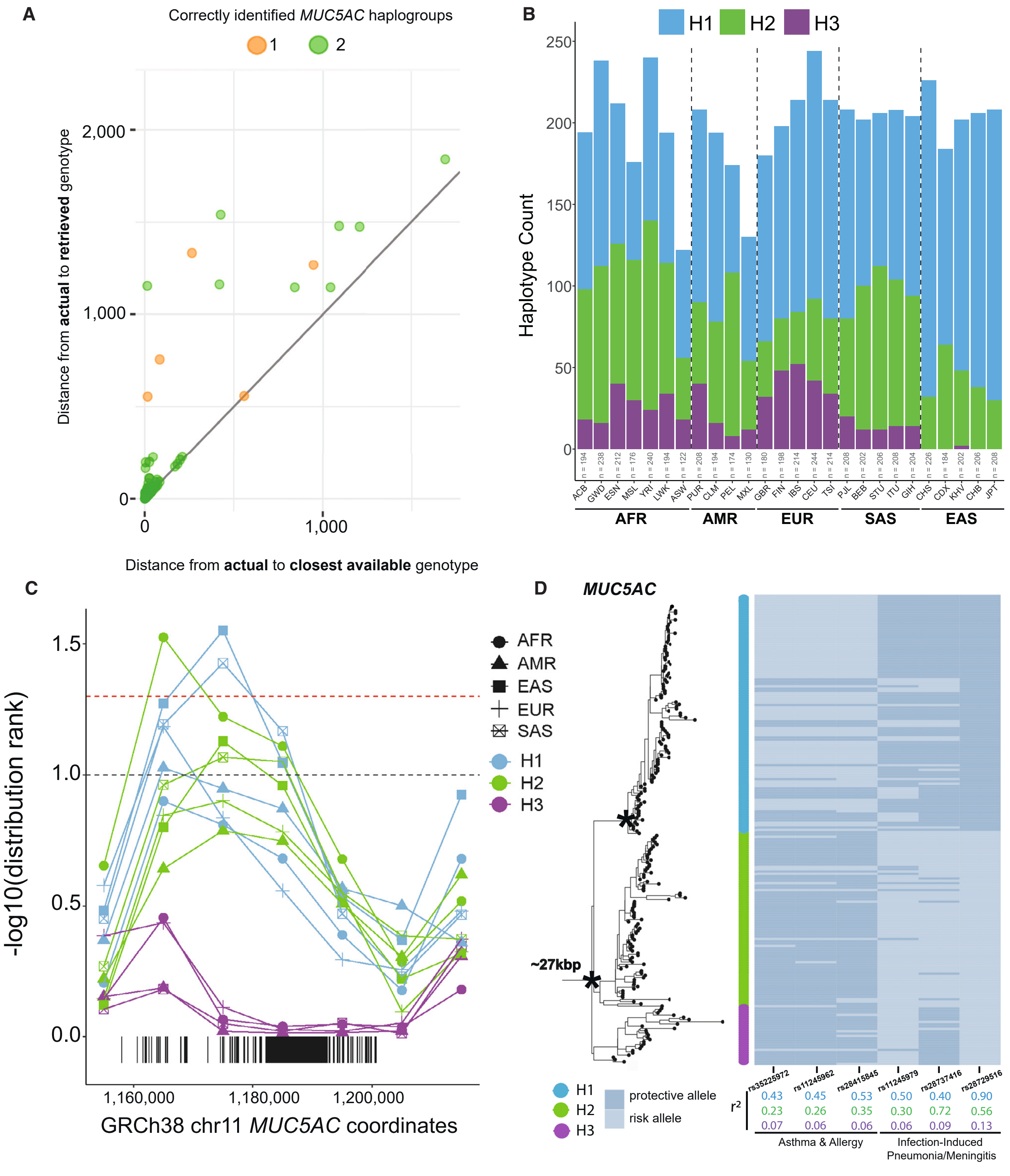

Figure 5. Genotyping of *MUC5AC* haplogroups with Locityper for population distributions and signatures of positive selection
(A) Locityper leave-one-out results comparing edit distances between actual and retrieved genotype (predicted from Locityper) versus edit distances between actual and closest possible genotype (best possible reference genotype from a multiple sequence alignment with true genotype) for *MUC5AC*. Dot color based on the number of haplotypes in diploid sample sets that were correctly genotyped.
(B) *MUC5AC* haplogroup frequencies across super populations and populations in the 1KG dataset from Locityper predictions.
(C) Distribution ranks of negative Tajima's D values across 10 kbp bins in the *MUC5AC* locus for genotyped haplogroups in each of the 1KG super populations. The dashed black line corresponds to the 10% distribution rank and the dashed red line corresponds to the 5% distribution rank. The three values above the dashed red line pass permutation testing and multiple testing correction.
(D) Six GWAS risk and protective alleles mapped to the *MUC5AC* phylogeny. SNPs grouped based on disease association and squared correlations color coded based on haplogroup partitioning.

Table 3. Tajima's D statistic for *MUC5AC* stratified by Locityper haplogroups in the 1KG

				GRCh38 chromosome 11 bin						
Population	Gene	Haplogroup	–	1,150,000	1,160,000	1,170,000	1,180,000[a]	1,190,000[a]	1,200,000	1,210,000
AFR	*MUC5AC*	H1	Tajima's D	−0.54	−1.36	−1.29	−1.18	−0.87	−0.47	−1.18
			# SNPs	156	215	321	205	197	185	131
		H2	Tajima's D	−1.17	−1.75[c,f]	−1.58[b]	−1.51[b]	−1.19	−0.71	−1.03
			# SNPs	176	214	332	214	199	198	142
		H3	Tajima's D	−0.19	−0.80	0.19	0.42[d]	0.34[d]	0.45[d]	−0.30
			# SNPs	134	166	261	157	160	157	104
AMR	*MUC5AC*	H1	Tajima's D	−0.86	−1.67[b]	−1.60	−1.53	−1.18	−1.08	−0.84
			# SNPs	121	155	244	165	155	141	78
		H2	Tajima's D	−0.21	−1.09	−1.29	−1.24	−0.88	−0.45	−1.06
			# SNPs	85	109	193	116	117	106	78
		H3	Tajima's D	0.42	0.28	1.61[e,f]	1.79[e,f]	1.72[e,f]	1.58[e,f]	−0.10
			# SNPs	64	70	142	94	93	69	50
EAS	*MUC5AC*	H1	Tajima's D	−0.67	−1.78[b]	−1.99[c,f]	−1.55[b]	−0.78	−0.41	−1.41
			# SNPs	84	138	216	102	84	77	77
		H2	Tajima's D	0.81	−1.08	−1.53[b]	−1.32	−0.54	0.32	−0.02
			# SNPs	64	112	198	107	99	77	54
		H3	Tajima's D	NA	NA	NA	NA	NA	NA	NA
			# SNPs	NA	NA	NA	NA	NA	NA	NA
EUR	*MUC5AC*	H1	Tajima's D	−0.84	−1.62[b]	−1.23	−0.81	−0.22	−0.11	−0.66
			# SNPs	97	157	257	150	125	101	77
		H2	Tajima's D	0.83	−0.93	−1.02	−0.83	−0.40	1.13	0.16
			# SNPs	69	105	157	109	104	73	54
		H3	Tajima's D	−0.16	−0.28	0.86	1.75[d]	1.93[e,f]	1.38	−0.13
			# SNPs	75	90	188	110	103	80	51
SAS	*MUC5AC*	H1	Tajima's D	−0.71	−1.66[b]	−1.85[c,f]	−1.64[b]	−0.74	−0.17	−0.74
			# SNPs	108	159	257	153	123	111	82
		H2	Tajima's D	−0.15	−1.36	−1.48[b]	−1.46[b]	−0.78	−0.46	−0.42
			# SNPs	97	142	220	140	113	109	72
		H3	Tajima's D	1.20	0.81	1.65	2.13[e,f]	1.62	2.39[e,f]	0.27
			# SNPs	47	58	135	82	90	54	35

Bin sizes of 10 kbp were used to compare values to the autosome-wide distribution per population and per haplogroup in the 1KG cohort.[19]
[a]Corresponds to bin containing VNTR sequence.
[b]Bottom 10% of autosome-wide Tajima's D values.
[c]Bottom 5% of autosome-wide Tajima's D values.
[d]Top 10% of autosome-wide Tajima's D values.
[e]Top 5% of autosome-wide Tajima's D values.
[f]Significant at $\alpha = 0.05$ after permutation testing.

Discussion

Using numerous high-quality long-read genome assemblies, we performed a population-level genetic survey of *MUC5AC* and *MUC5B* structural polymorphism. The protein-coding VNTRs of both loci have precluded and complicated the study of these genes from short-read WGS datasets. Initial efforts to resolve *MUC5AC* and *MUC5B* using long-read sequencing have relied on platforms with higher error rates and have been limited to a few individuals ($n = 4$)[7]; however, recent advances in long-read sequencing technologies and *de novo* genome assembly algorithms[11,15,16] have made complete characterization of the genes possible.[9,10] These analyses open a path to improved understanding of how mucin structural variants contribute to health and disease.

While our results recapitulate the long-held belief that *MUC5B* is less variable than other secreted mucins,[54] they refute the hypothesis that *MUC5B* is intolerant of structural changes, as we have identified structural variants of likely functional consequence among Africans. This is perhaps not surprising given the greater genetic diversity expected among Africans.[58] This variation has likely been missed because most studies of *MUC5B* have been conducted within European populations (e.g., *MUC5B* promoter polymorphism[50]). It is thus important that initiatives from consortia like the HGSVC,[9] HPRC,[10] and *All of Us*[49] broadly survey individuals of diverse genetic ancestry with long-read sequencing.

In contrast to *MUC5B*, we uncovered extensive aa composition and size variation within *MUC5AC*. This difference may be related to their varying functional roles; while *MUC5B* is ubiquitously and constitutively expressed in the airways, *MUC5AC* is overexpressed in the nasopharynx and is highly responsive to inflammation.[59] *MUC5AC* has likely evolved independently from *MUC5B* to respond to a wider variety of pathogenic challenges.[1]

Our comparative analyses with NHPs also indicates that VNTR length has generally decreased in the human lineage over the course of ape evolution for both genes. Increased VNTR length and subsequent glycosylation is predicted to enhance the interaction of the mucins with water,[58] thereby altering the mucus's biophysical properties.[60] Additionally, an increase in the number of cys domains may enhance non-covalent self-interactions that make the gel impermeable.[61] It is possible that longer variants of both mucins contribute to pathogenic changes in the viscoelastic properties of mucus in disease phenotypes, such as asthma and cystic fibrosis. In this regard, it is noteworthy that respiratory disease is a particularly pervasive problem affecting NHPs in captivity[62]; therefore, the reduction in overall VNTR length (especially in H1 and H2 haplogroups) may have been particularly adaptive in humans. Because of our detailed curation of many *MUC5AC* and *MUC5B* human haplotypes,[9,10,19] further experimental work uncovering how length variation in both loci imparts functional differences is now possible.

Within the human population, we distinguish three major *MUC5AC* haplogroups (H1–H3) that generally correlate with VNTR length (H1 encoding the shortest and H3 the longest molecules; Figure 1). The longer haplogroup variants are depleted among genomes of East Asian descent. We observe a signature of positive selection in East Asians, as evidenced by an excess of rare variants (Tajima's D) and extended LD. While this could be in part due to recent population bottlenecks or rapid population expansion in East Asians,[63] our genome-wide LD survey places *MUC5AC* block length in the top 5% (Figure 4). These findings may be relevant to the decreased prevalence of asthma in individuals of Asian descent,[64,65] although many other mitigating factors, such as environmental exposures,[61] play an important role.

We leveraged the LD and structural differences present within the 206 assembled haplotypes of *MUC5AC* and *MUC5B* to genotype short-read WGS data. Using the recently developed program Locityper, we estimate a high degree of genotyping accuracy (∼95% based on leave-one-out experiments). Applying Locityper to the high-coverage WGS data generated from 2,600 1KG samples[19] confirms the striking population stratification and positive selection signature among East Asian populations (Figure 5). Given the importance of *MUC5AC* as a genetic modifier of epithelial diseases like cystic fibrosis[8] and asthma/allergy,[39–41] it will be critical to continue cataloging haplotype diversity and improving short-read genotyping assays at this locus using haplotype information.

Our study of the genetic diversity of *MUC5AC* suggests different forces of both balancing and positive selection may be operating. Unlike H1, where LD block size and Tajima's D suggest positive selection in East Asians, our analysis of H3 in Europeans provides preliminary evidence of heterozygote advantage based on significantly positive Tajima's D and deviation from Hardy-Weinberg equilibrium. The molecular basis for this is unknown, but it is interesting that a protective effect was suggested by PheWAS for macular/retinal degeneration and enriched in H3 heterozygotes (Tables S14–S21). It is feasible that H3 variants provide a protective function against ocular disease because *MUC5AC* expression has been previously associated with dry eye syndrome.[66,67] *MUC5AC* and *MUC5B* are expressed in epithelial tissues outside of the lungs, and the signatures of selection we have uncovered may be due to more than just lung traits. It will be critical to understand these biological nuances and control for population substructure in future association studies.

At a broader level, the strategy we have outlined is applicable to other mucin loci and structurally variable genes. There are numerous gene families with protein-encoding structural polymorphisms that have generally been excluded from surveys of genetic variation and disease. Some of these are already known, such as *LPA*[68] and *CYP2D6*,[69] while others are suggestive, such as *HRNR*.[70] Even for *MUC5AC* and *MUC5B*, over 100 assembled reference genomes are still insufficient to capture the extent of human genetic diversity at these dynamic loci. Additional haplotypes from long reads in the HPRC, HGSVC, and *All of Us,* as well as approaches that tag haplotypes (as opposed to single SNPs), are needed to facilitate further variant discovery, protein domain sequence curation, LD block structure analysis, and genetic associations with disease. Importantly, the resulting panels of sequence-resolved haplotypes and tools like Locityper could facilitate direct genotyping from short reads in large population cohorts like *All of Us* or the UK Biobank. As long-read sequencing methods continue to be optimized and become less expensive in the coming years, the importance of these more complex forms of human genetic variation will become realized.

Data and code availability

The assemblies generated for this project (not previously published by the HGSVC[9]) were uploaded and accessioned via NCBI Sequence Read Archive (SRA). Sample accession IDs can be found in Tables S1 and S2. This study used data from the *All of Us* Research Program Controlled Tier Dataset v7, available to authorized users on the Researcher Workbench.

Supplemental information

Supplemental information can be found online at https://doi.org/10.1016/j.ajhg.2024.06.007.

Acknowledgments

We thank Tonia Brown and Michelle Noyes for assistance in editing this manuscript. We thank the HGSVC for access to the underlying PacBio HiFi CCS reads for assembly of the *MUC5AC/MUC5B* locus and the Primate T2T Consortium (especially Kateryna Makova and Adam Phillippy) for access to the high-quality data ape genome assemblies via GenomeArk. We thank DongAhn Yoo, Mei Wu, Brian Browning, Devin Schweppe, and Nick Riley for their intellectual contributions to the experimental designs, statistical analyses, and visualizations contained in this manuscript. The human cell lines used for sequence and assembly were obtained from the NIGMS Human Genetic Cell Repository at the Coriell Institute for Medical Research (Tables S1 and S2). The human samples were part of the 1000 Genomes Project. All genome sequence data are thus consented for open access with no data use restrictions. We gratefully acknowledge *All of Us* participants for their contributions to this work and thank the National Institutes of Health's *All of Us* Research Program for making the participant data available for this study. This work was supported, in part, by US National Institutes of Health (NIH) grants HG002385, HG010169, and HG007497 to E.E.E. E.E.E. and J.D.B. are investigators of the Howard Hughes Medical Institute.

This article is subject to HHMI's Open Access to Publications policy. HHMI lab heads have previously granted a nonexclusive CC BY 4.0 license to the public and a sublicensable license to HHMI in their research articles. Pursuant to those licenses, the author-accepted manuscript of this article can be made freely available under a CC BY 4.0 license immediately upon publication.

Author contributions

E.G.P., P.H., and E.E.E. conceived and planned the experiments. W.K.O., P.H., and J.D.B. provided critical intellectual support during project design. General methodologies were conceived by E.G.P., P.H., A.S., and E.E.E. E.G.P. performed data curation and formal analyses. T.P. and T.M. supported E.G.P. with Locityper analyses and data visualizations. E.N., E.J.K., and P.N.V. conceived and performed the PheWAS analyses. W.T.H. and K.M.M. provided technical and scientific consultation. Data visualization was designed by E.G.P. and E.E.E. E.G.P. and E.E.E. wrote the original manuscript, with edits and reviews from all authors. All authors provided critical feedback that shaped the research and analysis outlined in this manuscript.

Declaration of interests

E.E.E. is a scientific advisory board (SAB) member of Variant Bio, Inc.

Received: March 15, 2024
Accepted: June 17, 2024
Published: July 10, 2024

References

1. Chatterjee, M., van Putten, J.P.M., and Strijbis, K. (2020). Defensive properties of mucin glycoproteins during respiratory infections—relevance for Sars-CoV-2. mBio *11*.
2. Wallace, L.E., Liu, M., van Kuppeveld, F.J., de Vries, E., and de Haan, C.A. (2021). Respiratory mucus as a virus-host range determinant. Trends Microbiol. *29*, 983–992.
3. Morrison, C.B., Markovetz, M.R., and Ehre, C. (2019). Mucus, mucins, and cystic fibrosis. Pediatr. Pulmonol. *54*, S84–S96.
4. Bergstrom, K.S.B., and Xia, L. (2013). Mucin-type O-glycans and their roles in intestinal homeostasis. Glycobiology *23*, 1026–1037.
5. Chaisson, M.J.P., Sanders, A.D., Zhao, X., Malhotra, A., Porubsky, D., Rausch, T., Gardner, E.J., Rodriguez, O.L., Guo, L., Collins, R.L., et al. (2019). Multi-platform discovery of haplotype-resolved structural variation in human genomes. Nat. Commun. *10*, 1784.
6. Logsdon, G.A., Vollger, M.R., and Eichler, E.E. (2020). Long-read human genome sequencing and its applications. Nat. Rev. Genet. *21*, 597–614.
7. Guo, X., Zheng, S., Dang, H., Pace, R.G., Stonebraker, J.R., Jones, C.D., Boellmann, F., Yuan, G., Haridass, P., Fedrigo, O., et al. (2014). Genome reference and sequence variation in the large repetitive central exon of human MUC5AC. Am. J. Respir. Cell Mol. Biol. *50*, 223–232.
8. Guo, X., Pace, R.G., Stonebraker, J.R., Commander, C.W., Dang, A.T., Drumm, M.L., Harris, A., Zou, F., Swallow, D.M., Wright, F.A., et al. (2011). Mucin variable number tandem repeat polymorphisms and severity of cystic fibrosis lung disease: significant association with MUC5AC. PLoS One *6*, e25452.
9. Ebert, P., Audano, P.A., Zhu, Q., Rodriguez-Martin, B., Porubsky, D., Bonder, M.J., Sulovari, A., Ebler, J., Zhou, W., Serra Mari, R., et al. (2021). Haplotype-resolved diverse human genomes and integrated analysis of structural variation. Science *372*.
10. Liao, W.W., Asri, M., Ebler, J., Doerr, D., Haukness, M., Hickey, G., Lu, S., Lucas, J.K., Monlong, J., Abel, H.J., et al. (2023). A draft human pangenome reference. Nature *617*, 312–324.
11. Cheng, H., Concepcion, G.T., Feng, X., Zhang, H., and Li, H. (2021). Haplotype-resolved de novo assembly using phased assembly graphs with hifiasm. Nat. Methods *18*, 170–175.
12. Vollger, M.R., Dishuck, P.C., Sorensen, M., Welch, A.E., Dang, V., Dougherty, M.L., Graves-Lindsay, T.A., Wilson, R.K., Chaisson, M.J.P., and Eichler, E.E. (2018). Long-read sequence and assembly of segmental duplications. Nat. Methods *16*, 88–94.
13. Mao, Y., Harvey, W.T., Porubsky, D., Munson, K.M., Hoekzema, K., Lewis, A.P., Audano, P.A., Rozanski, A., Yang, X., Zhang, S., et al. (2024). Structurally divergent and recurrently mutated regions of primate genomes. Cell *187*, 1547–1562.e13.

14. Makova, K.D., Pickett, B.D., Harris, R.S., Hartley, G.A., Cechova, M., Pal, K., Nurk, S., Yoo, D., Li, Q., Hebbar, P., et al. (2024). The complete sequence and comparative analysis of ape sex chromosomes. Nature *630*, 401–411.
15. Rautiainen, M., Nurk, S., Walenz, B.P., Logsdon, G.A., Porubsky, D., Rhie, A., Eichler, E.E., Phillippy, A.M., and Koren, S. (2023). Telomere-to-telomere assembly of diploid chromosomes with Verkko. Nat. Biotechnol. *41*, 1474–1482.
16. Nurk, S., Koren, S., Rhie, A., Rautiainen, M., Bzikadze, A.V., Mikheenko, A., Vollger, M.R., Altemose, N., Uralsky, L., Gershman, A., et al. (2022). The complete sequence of a human genome. Science *376*, 44–53.
17. Li, H. (2018). Minimap2: pairwise alignment for nucleotide sequences. Bioinformatics *34*, 3094–3100.
18. Frankish, A., Carbonell-Sala, S., Diekhans, M., Jungreis, I., Loveland, J.E., Mudge, J.M., Sisu, C., Wright, J.C., Arnan, C., Barnes, I., et al. (2023). GENCODE: reference annotation for the human and mouse genomes in 2023. Nucleic Acids Res. *51*, D942–D949.
19. Byrska-Bishop, M., Evani, U.S., Zhao, X., Basile, A.O., Abel, H.J., Regier, A.A., Corvelo, A., Clarke, W.E., Musunuri, R., Nagulapalli, K., et al. (2022). High-coverage whole-genome sequencing of the expanded 1000 Genomes Project cohort including 602 trios. Cell *185*, 3426–3440.e19.
20. Katoh, K., and Standley, D.M. (2013). MAFFT multiple sequence alignment software version 7: improvements in performance and usability. Mol. Biol. Evol. *30*, 772–780.
21. Waterhouse, A.M., Procter, J.B., Martin, D.M.A., Clamp, M., and Barton, G.J. (2009). Jalview Version 2—a multiple sequence alignment editor and analysis workbench. Bioinformatics *25*, 1189–1191.
22. Nguyen, L.T., Schmidt, H.A., Von Haeseler, A., and Minh, B.Q. (2015). IQ-TREE: a fast and effective stochastic algorithm for estimating maximum-likelihood phylogenies. Mol. Biol. Evol. *32*, 268–274.
23. Xu, S., Li, L., Luo, X., Chen, M., Tang, W., Zhan, L., Dai, Z., Lam, T.T., Guan, Y., and Yu, G. (2022). Ggtree: a serialized data object for visualization of a phylogenetic tree and annotation data. IMeta *1*, e56.
24. Minh, B.Q., Schmidt, H.A., Chernomor, O., Schrempf, D., Woodhams, M.D., Von Haeseler, A., and Lanfear, R. (2020). IQ-TREE 2: new models and efficient methods for phylogenetic inference in the genomic era. Mol. Biol. Evol. *37*, 1530–1534.
25. Dunsworth, H.M. (2010). Origin of the genus Homo. Evo. Edu. Outreach *3*, 353–366.
26. Rice, P., Longden, I., and Bleasby, A. (2000). EMBOSS: the European molecular biology open software suite. Trends Genet. *16*, 276–277.
27. Ho, S.B., Roberton, A.M., Shekels, L.L., Lyftogt, C.T., Niehans, G.A., and Toribara, N.W. (1995). Expression cloning of gastric mucin complementary DNA and localization of mucin gene expression. Gastroenterology *109*, 735–747.
28. Desseyn, J.L., Guyonnet-Dupérat, V., Porchet, N., Aubert, J.P., and Laine, A. (1997). Human mucin gene MUC5B, the 10.7-kb large central exon encodes various alternate subdomains resulting in a super-repeat: structural evidence for a 11p15.5 gene family. J. Biol. Chem. *272*, 3168–3178.
29. RR, S. (1958). A statistical method for evaluating systematic relationships. Univ. Kans. Sci. Bull. *38*, 1409–1438.
30. Benson, G. (1999). Tandem repeats finder: a program to analyze DNA sequences. Nucleic Acids Res. *27*, 573–580.
31. Bailey, T.L. (2021). STREME: accurate and versatile sequence motif discovery. Bioinformatics *37*, 2834–2840.
32. Dong, S.S., He, W.M., Ji, J.J., Zhang, C., Guo, Y., and Yang, T.L. (2021). LDBlockShow: a fast and convenient tool for visualizing linkage disequilibrium and haplotype blocks based on variant call format files. Briefings Bioinf. *22*.
33. Slatkin, M. (2008). Linkage disequilibrium—understanding the evolutionary past and mapping the medical future. Nat. Rev. Genet. *9*, 477–485.
34. Purcell, S., Neale, B., Todd-Brown, K., Thomas, L., Ferreira, M.A., Bender, D., Maller, J., Sklar, P., de Bakker, P.I., Daly, M.J., et al. (2007). PLINK: a tool set for whole-genome association and population-based linkage analyses. Am. J. Hum. Genet. *81*, 559–575.
35. Gabriel, S.B., Schaffner, S.F., Nguyen, H., Moore, J.M., Roy, J., Blumenstiel, B., Higgins, J., Defelice, M., Lochner, A., Faggart, M., et al. (2002). The structure of haplotype blocks in the human genome. Science *296*, 2225–2229.
36. Rousseau, K., Byrne, C., Griesinger, G., Leung, A., Chung, A., Hill, A.S., and Swallow, D.M. (2007). Allelic association and recombination hotspots in the mucin gene (MUC) complex on chromosome 11p15. 5. Ann. Hum. Genet. *71*, 561–569.
37. Tajima, F. (1983). Evolutionary relationship of DNA sequences in finite populations. Genetics *105*, 437–460.
38. MacArthur, J., Bowler, E., Cerezo, M., Gil, L., Hall, P., Hastings, E., Junkins, H., McMahon, A., Milano, A., Morales, J., et al. (2017). The new NHGRI-EBI Catalog of published genome-wide association studies (GWAS Catalog). Nucleic Acids Res. *45*, D896–D901.
39. Valette, K., Li, Z., Bon-Baret, V., Chignon, A., Bérubé, J.C., Eslami, A., Lamothe, J., Gaudreault, N., Joubert, P., Obeidat, M., et al. (2021). Prioritization of candidate causal genes for asthma in susceptibility loci derived from UK Biobank. Commun. Biol. *4*, 700.
40. Vuckovic, D., Bao, E.L., Akbari, P., Lareau, C.A., Mousas, A., Jiang, T., Chen, M.H., Raffield, L.M., Tardaguila, M., Huffman, J.E., et al. (2020). The polygenic and monogenic basis of blood traits and diseases. Cell *182*, 1214–1231.e11.
41. Ferreira, M.A., Mathur, R., Vonk, J.M., Szwajda, A., Brumpton, B., Granell, R., Brew, B.K., Ullemar, V., Lu, Y., Jiang, Y., et al. (2019). Genetic architectures of childhood-and adult-onset asthma are partly distinct. Am. J. Hum. Genet. *104*, 665–684.
42. Reay, W.R., Geaghan, M.P., Agee, M., Alipanahi, B., Bell, R.K., Bryc, K., Elson, S.L., Fontanillas, P., Furlotte, N.A., Hicks, B., et al. (2022). The genetic architecture of pneumonia susceptibility implicates mucin biology and a relationship with psychiatric illness. Nat. Commun. *13*, 3756.
43. Tian, C., Hromatka, B.S., Kiefer, A.K., Eriksson, N., Noble, S.M., Tung, J.Y., and Hinds, D.A. (2017). Genome-wide association and HLA region fine-mapping studies identify susceptibility loci for multiple common infections. Nat. Commun. *8*, 599.
44. Sabo, M.C., Thuong, N.T.T., Chang, X., Ardiansyah, E., Tram, T.T.B., Hai, H.T., Nghia, H.D.T., Bang, N.D., Dian, S., Ganiem, A.R., et al. (2023). MUC5AC genetic variation is associated with tuberculous meningitis cerebral spinal fluid cytokine responses and mortality. JID (J. Infect. Dis.) *228*, 343–352.
45. Prodanov, T., Plender, E.G., Seebohm, G., Meuth, S.G., Eichler, E.E., and Marschall, T. (2024). Locityper: targeted genotyping of complex polymorphic genes. Preprint at bioRxiv. https://doi.org/10.1101/2024.05.03.592358.
46. Danecek, P., Bonfield, J.K., Liddle, J., Marshall, J., Ohan, V., Pollard, M.O., Whitwham, A., Keane, T., McCarthy, S.A.,

Davies, R.M., and Li, H. (2021). Twelve years 5 of SAMtools and BCFtools. GigaScience *10*.

47. Marçais, G., and Kingsford, C. (2011). A fast, lock-free approach for efficient parallel counting of occurrences of k-mers. Bioinformatics *27*, 764–770.
48. Sahlin, K. (2022). Strobealign: flexible seed size enables ultra-fast and accurate read alignment. Genome Biol. *23*, 260.
49. Bick, A.G., Metcalf, G.A., Mayo, K.R., Lichtenstein, L., Rura, S., Carroll, R.J., Musick, A., Linder, J.E., Jordan, I.K., Nagar, S.D., et al. (2024). Genomic data in the All of Us Research Program. Nature *627*, 340–346.
50. Seibold, M.A., Wise, A.L., Speer, M.C., Steele, M.P., Brown, K.K., Loyd, J.E., Fingerlin, T.E., Zhang, W., Gudmundsson, G., Groshong, S.D., et al. (2011). A common MUC5B promoter polymorphism and pulmonary fibrosis. N. Engl. J. Med. *364*, 1503–1512.
51. O'Connell, B.C., and Tabak, L.A. (1993). A comparison of serine and threonine O-glycosylation by UDP-GaINAc: polypeptide N-acetylgalactosaminyltransferase. J. Dent. Res. *72*, 1554–1558.
52. Brockhausen, I., Schachter, H., and Stanley, P. (2009). O-GalNAc glycans. Essentials of Glycobiology, 2nd edition.
53. Ridley, C., Lockhart-Cairns, M.P., Collins, R.F., Jowitt, T.A., Subramani, D.B., Kesimer, M., Baldock, C., and Thornton, D.J. (2019). The C-terminal dimerization domain of the respiratory mucin MUC5B functions in mucin stability and intracellular packaging before secretion. J. Biol. Chem. *294*, 17105–17116.
54. Vinall, L.E., Hill, A.S., Pigny, P., Pratt, W.S., Toribara, N., Gum, J.R., Kim, Y.S., Porchet, N., Aubert, J.P., and Swallow, D.M. (1998). Variable number tandem repeat polymorphism of the mucin genes located in the complex on 11p15. 5. Hum. Genet. *102*, 357–366.
55. Kageyama-Yahara, N., Yamamichi, N., Takahashi, Y., Takeuchi, C., Matsumoto, Y., Sakaguchi, Y., and Koike, K. (2019). Tandem repeats of the 5′ flanking region of human MUC5AC have a role as a novel enhancer in MUC5AC gene expression. Biochemistry and Biophysics Reports *18*, 100632.
56. Wang, C., Wang, J., Liu, Y., Guo, X., and Zhang, C. (2014). MUC5AC upstream complex repetitive region length polymorphisms are associated with susceptibility and clinical stage of gastric cancer. PLoS One *9*, e98327.
57. Altman, M.C., Flynn, K., Rosasco, M.G., Dapas, M., Kattan, M., Lovinsky-Desir, S., O'Connor, G.T., Gill, M.A., Gruchalla, R.S., Liu, A.H., et al. (2021). Inducible expression quantitative trait locus analysis of the MUC5AC gene in asthma in urban populations of children. J. Allergy Clin. Immunol. *148*, 1505–1514.
58. 1000 Genomes Project Consortium (2015). A global reference for human genetic variation. Nature *526*, 68.
59. Singanayagam, A., Footitt, J., Marczynski, M., Radicioni, G., Cross, M.T., Finney, L.J., Trujillo-Torralbo, M.B., Calderazzo, M., Zhu, J., Aniscenko, J., et al. (2022). Airway mucins promote immunopathology in virus-exacerbated chronic obstructive pulmonary disease. J. Clin. Invest. *132*.
60. Cone, R.A. (2009). Barrier properties of mucus. Adv. Drug Deliv. Rev. *61*, 75–85.
61. Demouveaux, B., Gouyer, V., Robbe-Masselot, C., Gottrand, F., Narita, T., and Desseyn, J.L. (2019). Mucin CYS domain stiffens the mucus gel hindering bacteria and spermatozoa. Sci. Rep. *9*, 16993.
62. Lowenstine, L.J., and Osborn, K.G. (2012). Respiratory System Diseases of Nonhuman Primates (Nonhuman Primates in Biomedical Research), p. 413.
63. Cai, X., Qin, Z., Wen, B., Xu, S., Wang, Y., Lu, Y., Wei, L., Wang, C., Li, S., Huang, X., et al. (2011). Human migration through bottlenecks from Southeast Asia into East Asia during Last Glacial Maximum revealed by Y chromosomes. PLoS One *6*, e24282.
64. Pate, C.A., Zahran, H.S., Qin, X., Johnson, C., Hummelman, E., and Malilay, J. (2021). Asthma surveillance—United States, 2006–2018. MMWR. Surveill. Summ. *70*, 1–32.
65. Song, P., Adeloye, D., Salim, H., Dos Santos, J.P., Campbell, H., Sheikh, A., and Rudan, I. (2022). Global, regional, and national prevalence of asthma in 2019: a systematic analysis and modelling study. J. Glob. Health *12*, 04052.
66. Bhattacharya, D., Yu, L., and Wang, M. (2017). Expression patterns of conjunctival mucin 5AC and aquaporin 5 in response to acute dry eye stress. PLoS One *12*, e0187188.
67. Corrales, R.M., Narayanan, S., Fernández, I., Mayo, A., Galarreta, D.J., Fuentes-Páez, G., Chaves, F.J., Herreras, J.M., and Calonge, M. (2011). Ocular mucin gene expression levels as biomarkers for the diagnosis of dry eye syndrome. Invest. Ophthalmol. Vis. Sci. *52*, 8363–8369.
68. Schmidt, K., Noureen, A., Kronenberg, F., and Utermann, G. (2016). Structure, function, and genetics of lipoprotein (a). JLR (J. Lipid Res.) *57*, 1339–1359.
69. Dalton, R., Lee, S.B., Claw, K.G., Prasad, B., Phillips, B.R., Shen, D.D., Wong, L.H., Fade, M., McDonald, M.G., Dunham, M.J., et al. (2020). Interrogation of *<scp>CYP</scp>2D6* Structural Variant Alleles Improves the Correlation Between *<scp>CYP</scp>2D6* Genotype and CYP2D6-Mediated Metabolic Activity. Clinical Translational Sci. *13*, 147–156.
70. Lu, T.Y., Smaruj, P.N., Fudenberg, G., Mancuso, N., and Chaisson, M.J. (2023). The motif composition of variable number tandem repeats impacts gene expression. Genome Res. *33*, 511–524.

The American Journal of Human Genetics, Volume *111*

Supplemental information

Structural and genetic diversity in the secreted mucins *MUC5AC* and *MUC5B*

Elizabeth G. Plender, Timofey Prodanov, PingHsun Hsieh, Evangelos Nizamis, William T. Harvey, Arvis Sulovari, Katherine M. Munson, Eli J. Kaufman, Wanda K. O'Neal, Paul N. Valdmanis, Tobias Marschall, Jesse D. Bloom, and Evan E. Eichler

Note S1. Using tandem repeats finder, we discovered an 8-mer VNTR in intron 15 of *MUC5AC* with a canonical motif of TCACCCAC in all human haplotypes. Haplogroup 3 (H3) alleles harbor more copies of the repeat and a lower percent motif identity compared to haplogroup 1 and 2 alleles in humans. STREME (Sensitive, Thorough, Rapid, Enriched Motif Elicitation) analysis reveals five 24-mers that are exclusive to H3 alleles for genotyping this haplogroup (ACCATTCACTCACCCATTCACCCATTCACC, ACTCACCCACTCACCCATTCACCCATTCAC, ACTCACCCACTCACTCACTCACCTACTCAA, CAGTGGGTGATTGAGTGGGTGAATGGGTGA, and TAAGTTGAGTGAGTGAGGTGAGTGAGTGGA). The canonical 8-mer motif is exclusive to human haplotypes, with all NHPs featuring motif lengths of 12-28 nucleotides. Total VNTR copy number is also highly variable among the NHPs, leading to differences in total sequence length for intron 15 across haplotypes (Fig. S5).

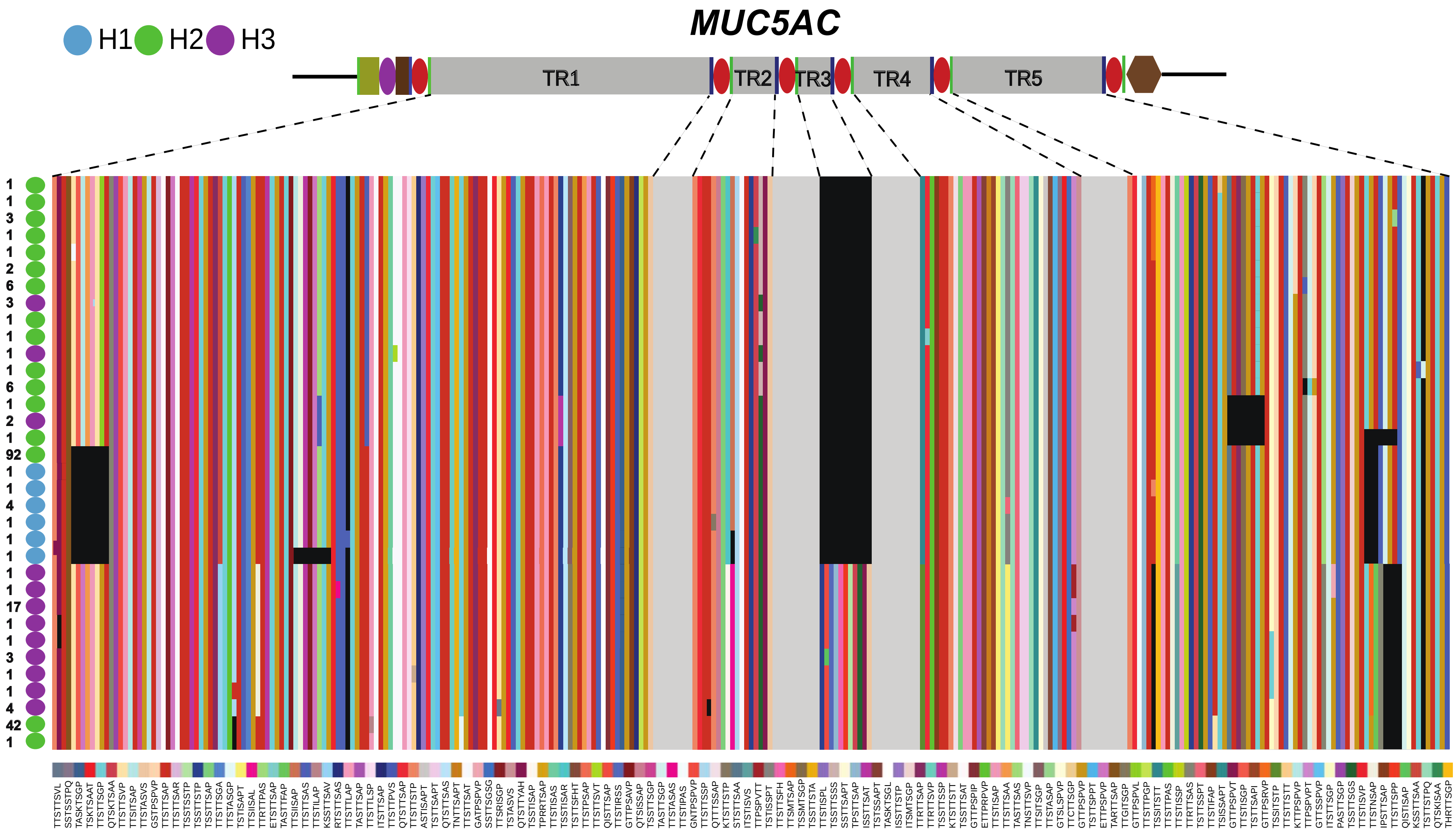
H1
H2
H3
MUC5AC
TR1
TR2
TR3
TR4
TR5
1
1
3
1
1
2
6
3
1
1
1
1
6
1
2
1
92
1
1
4
1
1
1
1
1
17
1
1
3
1
1
4
42
1

Figure S1. MUC5AC protein VNTR motif utilization across 206 human haplotypes. MUC5AC protein 8-mer variation plots across the five possible VNTR domains. Domain plot height corresponds to the total number of unique alleles across the large central exon. Numbers per unique allele correspond to the number of haplotypes that are matching in motif composition across all domains in linear sequence space. Black boxes indicate an absence of sequence. Gray represents cys domain sequence between distinct VNTR domains. Alleles are ordered vertically by hierarchical clustering of motif composition. Motifs are shifted in sequence space based on the same identity (indicating linear shifts of individual motifs).

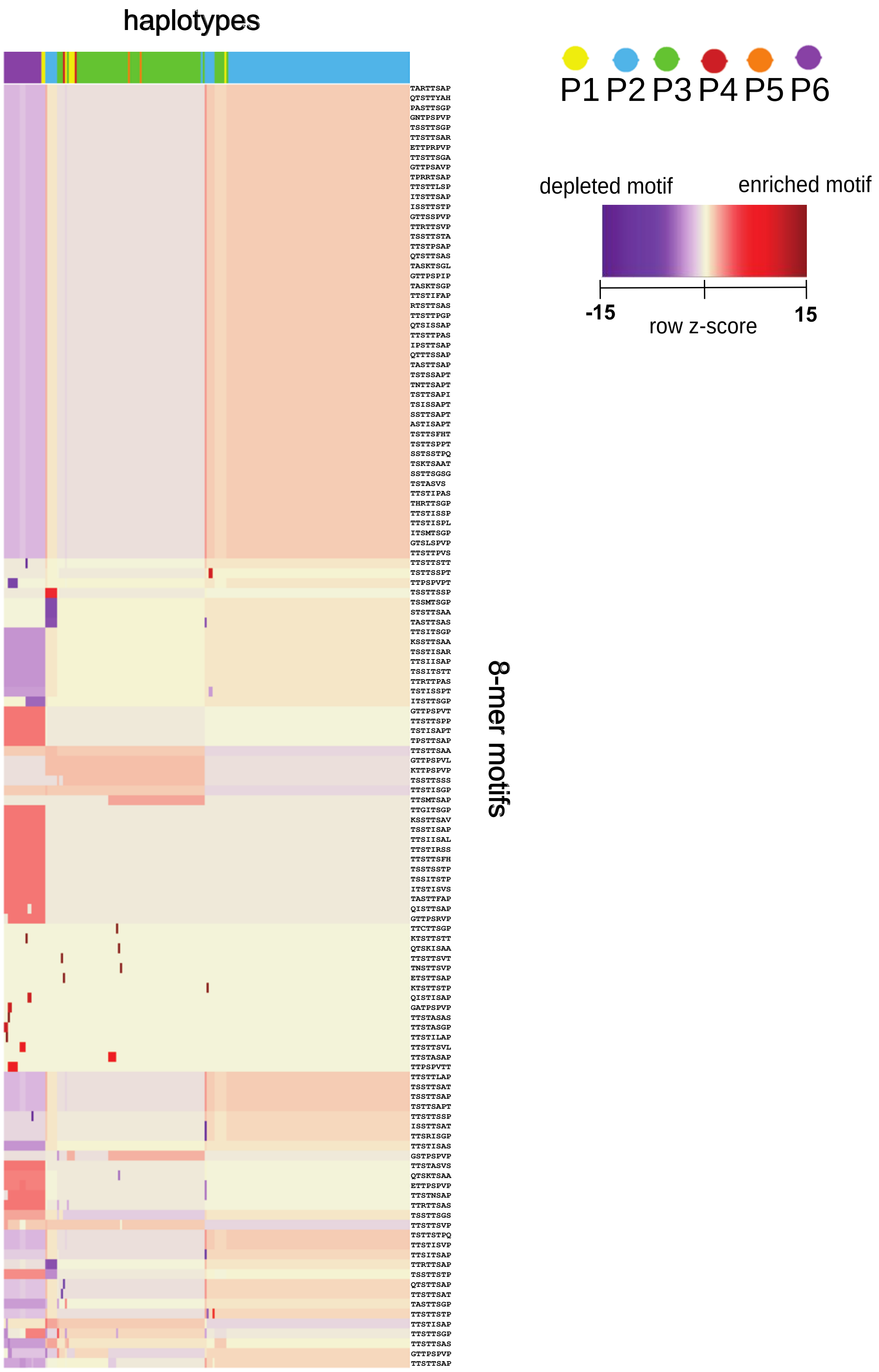

Figure S2. Extended version of Figure 1G.

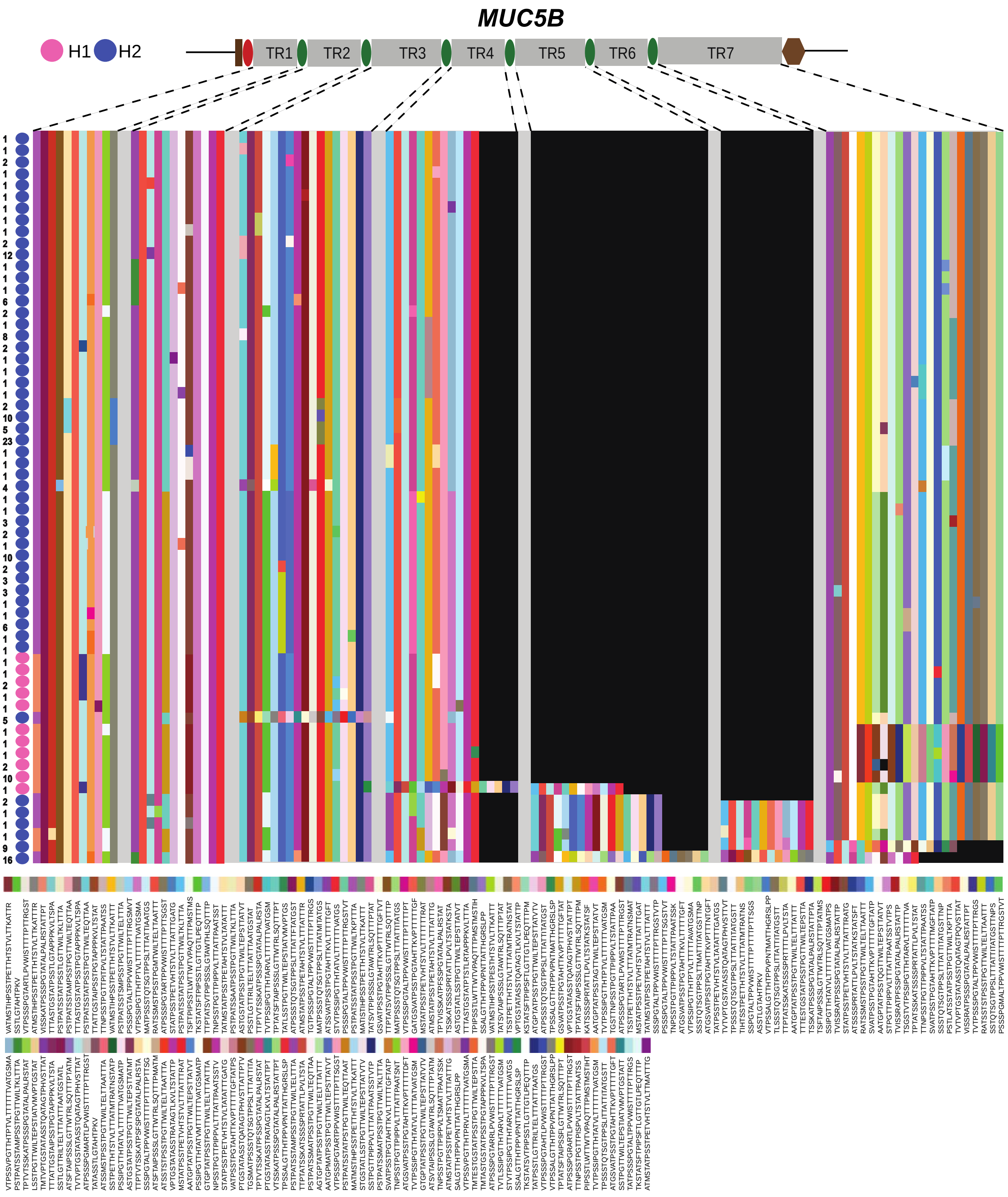
MUC5B
H1
H2
TR1
TR2
TR3
TR4
TR5
TR6
TR7

Figure S3. MUC5B protein VNTR motif utilization across 206 human haplotypes. MUC5B protein 29-mer variation plots across the five possible VNTR domains. Domain plot height corresponds to the total number of unique alleles across the large central exon. The numbers per unique allele correspond to the number of haplotypes that are matching in motif composition across all domains in linear sequence space. Black boxes indicate an absence of sequence. Gray represents cys domain sequence between distinct VNTR domains. Alleles are ordered vertically by hierarchical clustering of motif composition. Motifs are shifted in sequence space based on the same identity (indicating linear shifts of individual motifs).

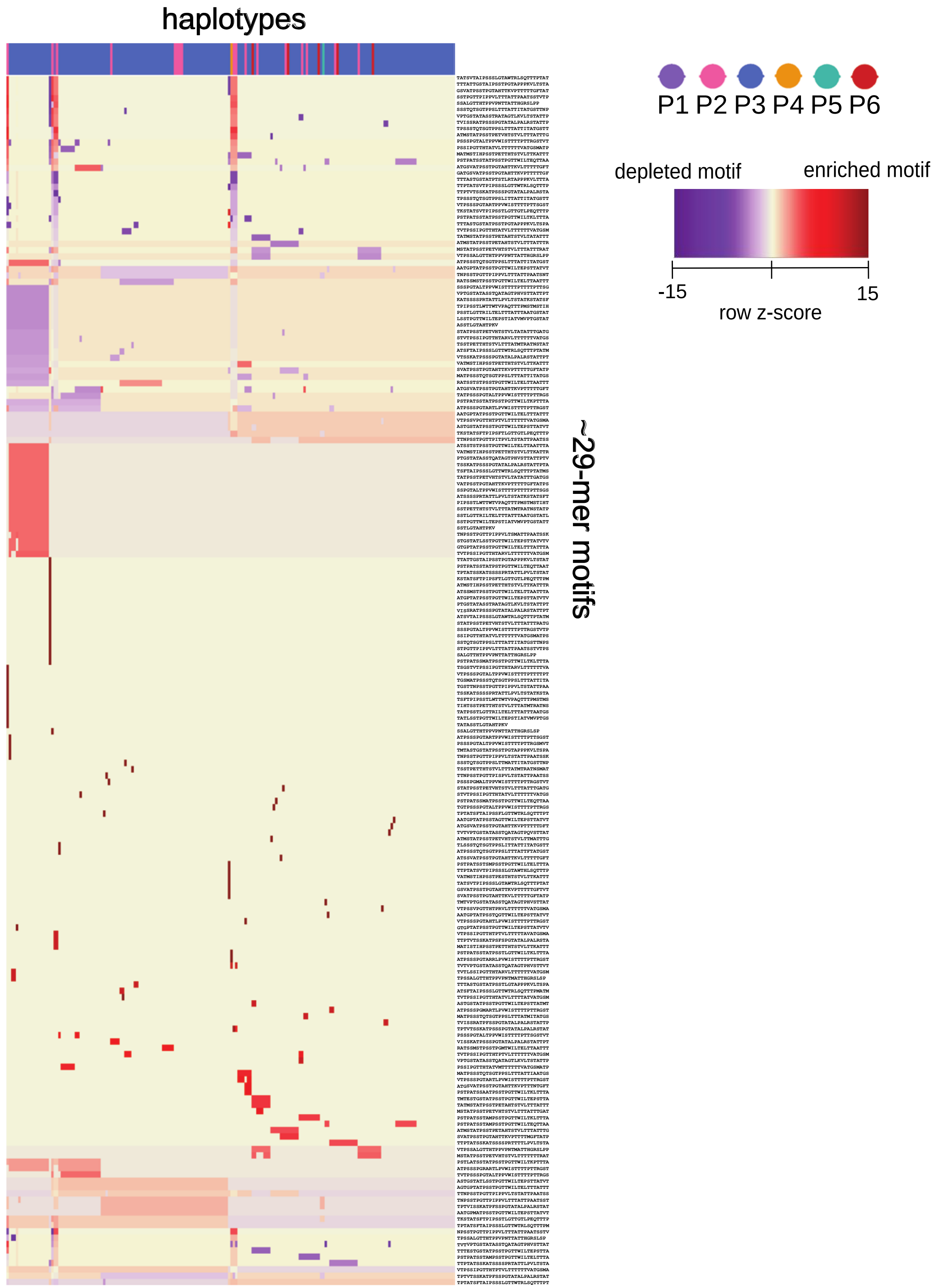

Figure S4. Extended version of Figure 2G.

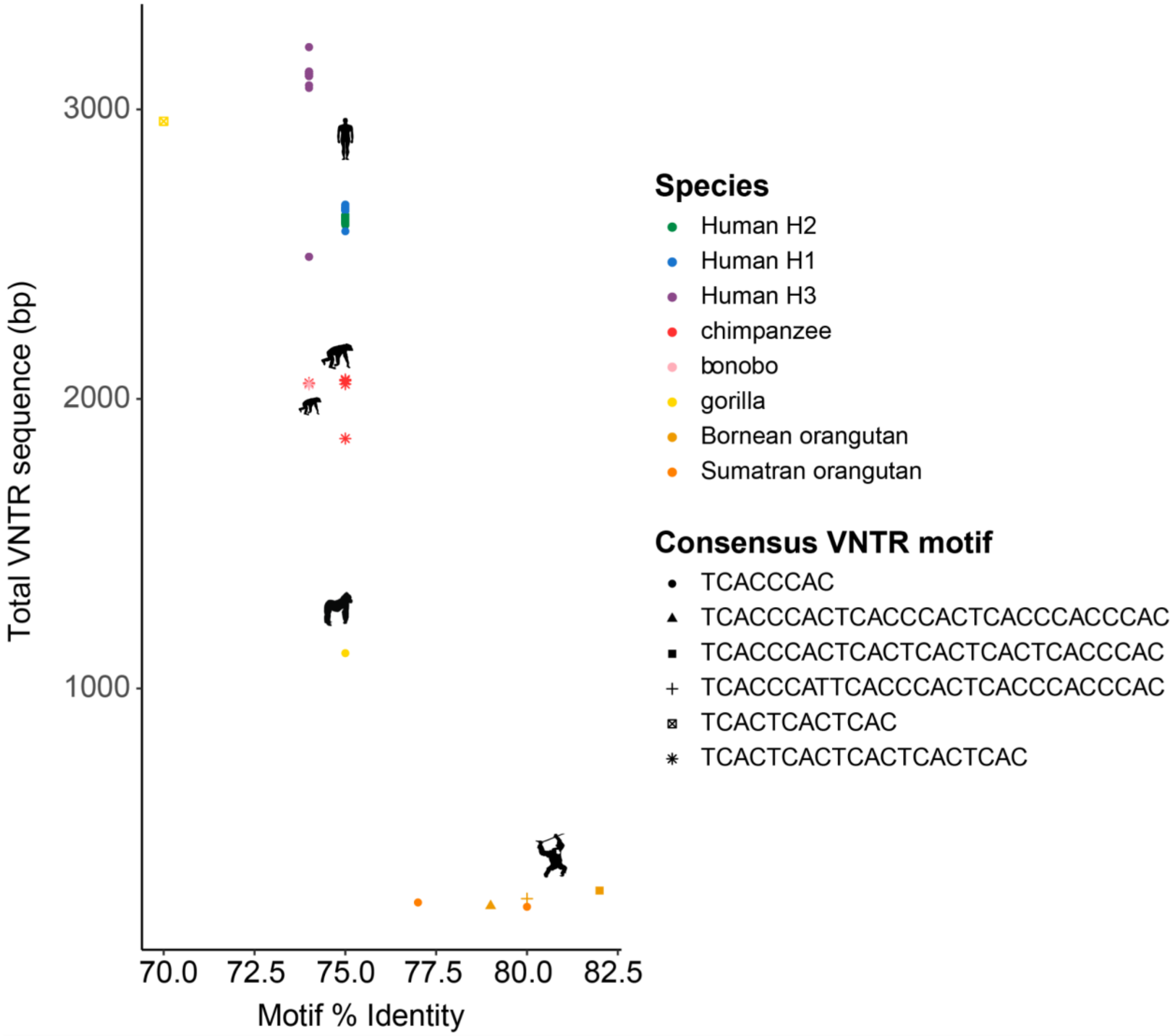

Figure S5. Intron 15 VNTR sequence characterization in *MUC5AC* for humans and nonhuman apes. Comparison of total VNTR sequence length (in base pairs) and canonical motif percent identity across 206 human haplotypes and at least two haplotypes per nonhuman ape species. Species/haplogroup identity coded by color and shape coded by motif.

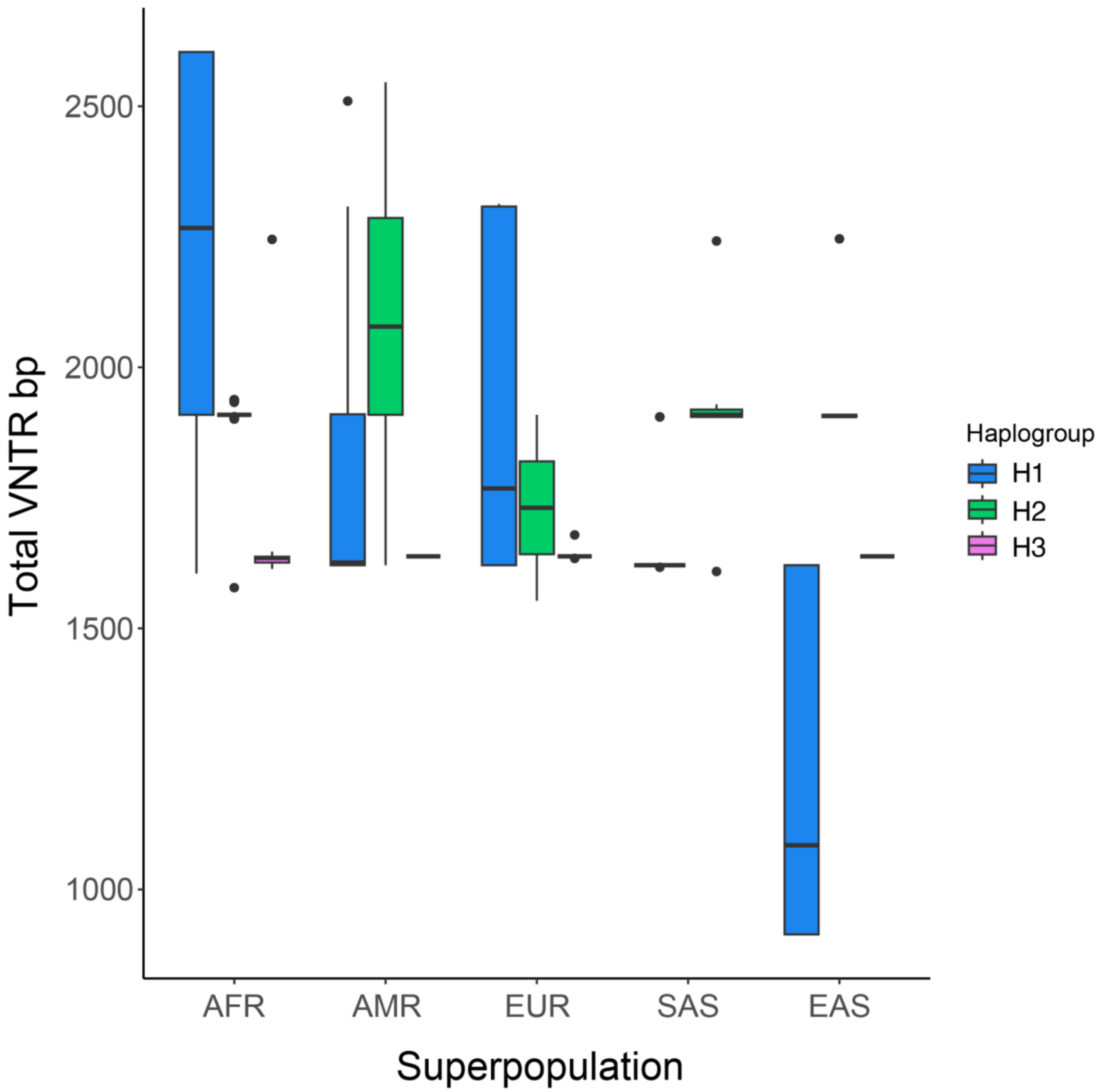

Figure S6. ***MUC5AC*** **VNTR enhancer polymorphism across human super populations in the HGSVC/HPRC sample set, stratified by haplogroup identity.** Total VNTR bp indicates the longest continuous track of degenerate 8-mer VNTR sequence in the enhancer region of *MUC5AC.* Haplogroups correspond to phylogenetic clades of the *MUC5AC* locus.

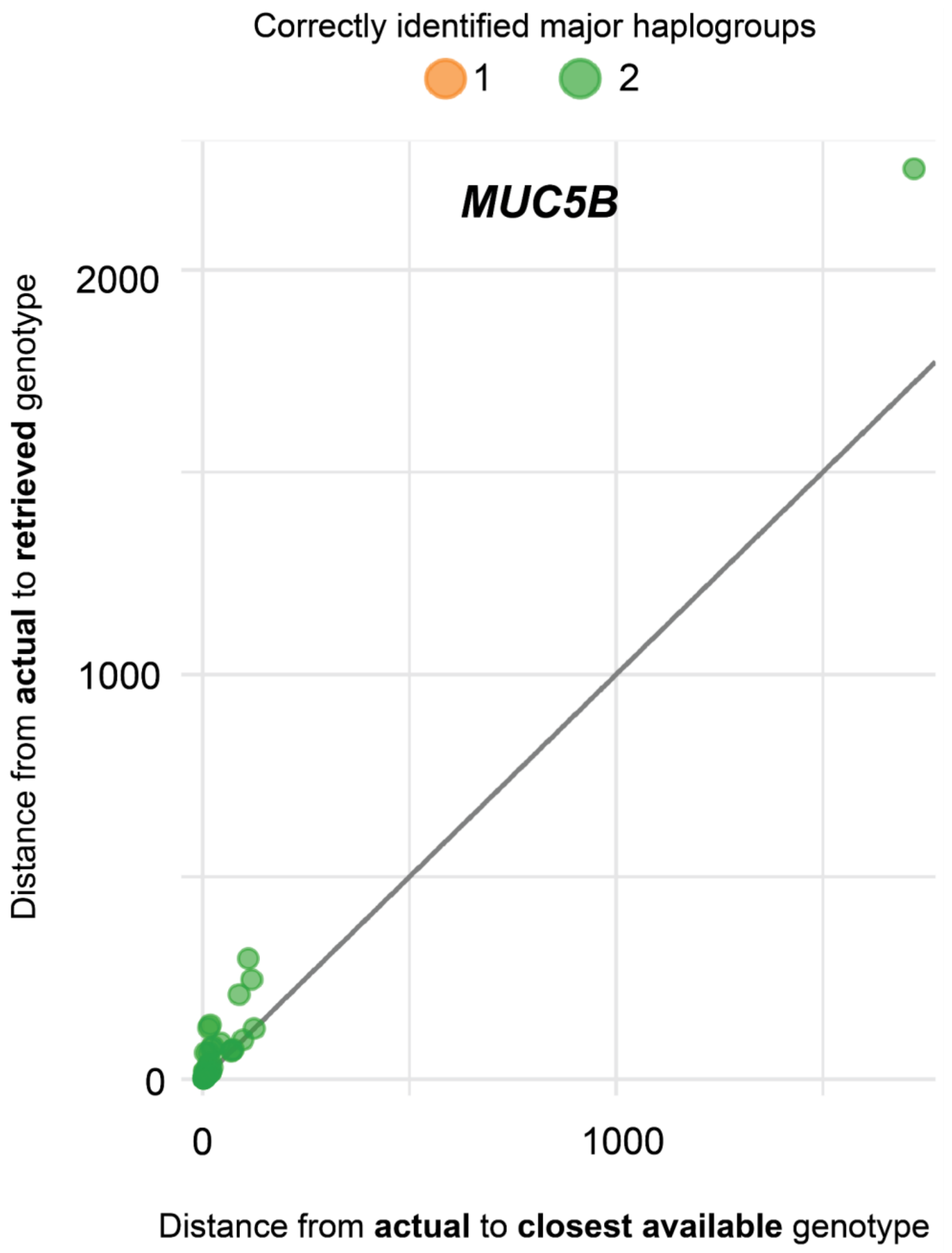

Figure S7. Genotyping accuracy of *MUC5B* haplogroups with Locityper. Locityper leave-one-out results comparing edit distances between actual and retrieved genotype (predicted from genotyper) versus edit distances between actual and closest possible genotype (best possible reference genotype from multiple sequence alignment with true genotype) for *MUC5B.* Dot color corresponds to the number of haplotypes in diploid sample sets that were correctly genotyped.

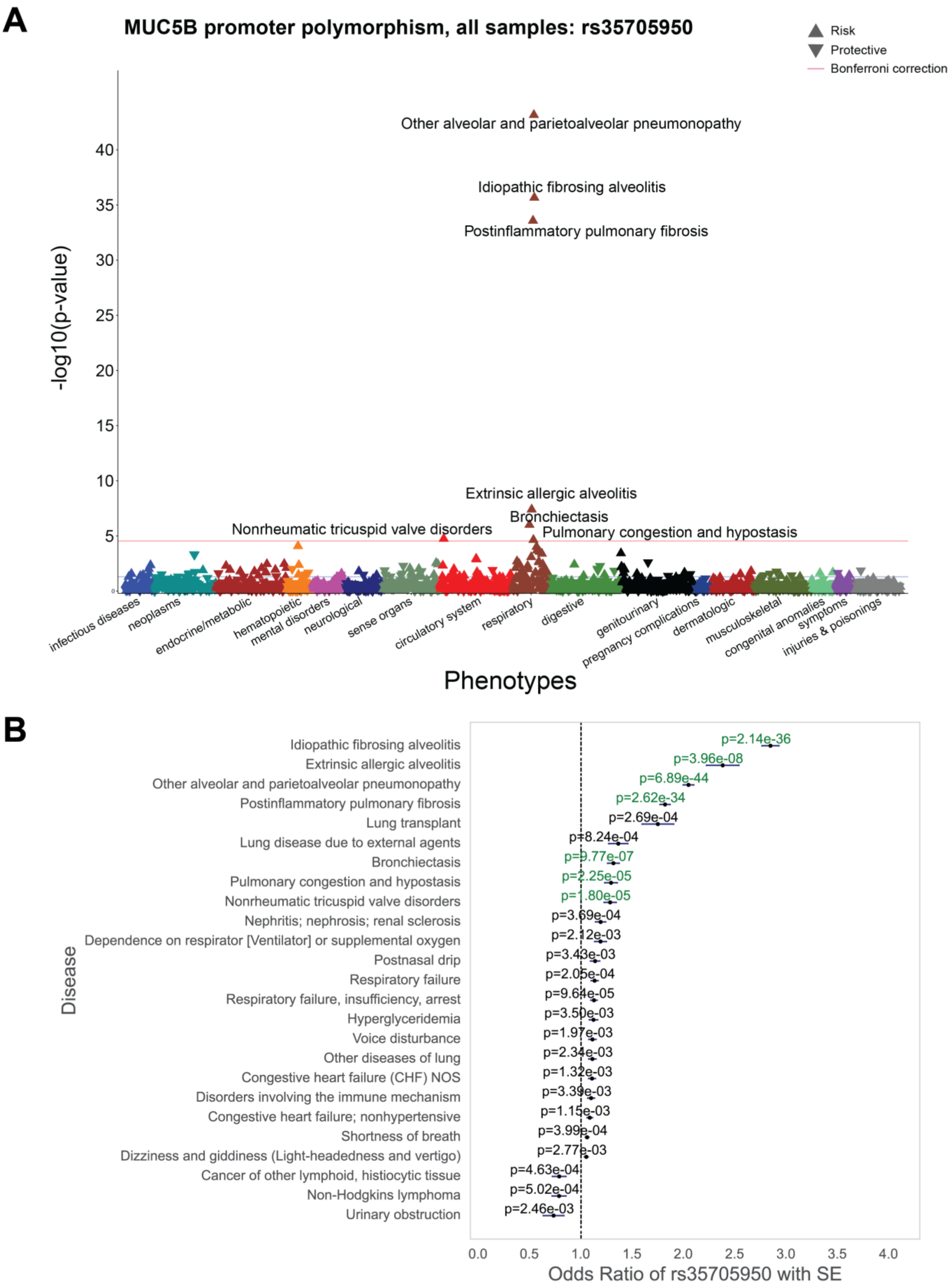
A
MUC5B promoter polymorphism, all samples: rs35705950
Risk
Protective
Bonferroni correction
-log10(p-value)
Other alveolar and parietoalveolar pneumonopathy
Idiopathic fibrosing alveolitis
Postinflammatory pulmonary fibrosis
Extrinsic allergic alveolitis
Bronchiectasis
Nonrheumatic tricuspid valve disorders
Pulmonary congestion and hypostasis
infectious diseases
neoplasms
endocrine/metabolic
hematopoietic
mental disorders
neurological
sense organs
circulatory system
respiratory
digestive
genitourinary
pregnancy complications
dermatologic
musculoskeletal
congenital anomalies
symptoms
injuries & poisonings
Phenotypes
B
Disease
Idiopathic fibrosing alveolitis
Extrinsic allergic alveolitis
Other alveolar and parietoalveolar pneumonopathy
Postinflammatory pulmonary fibrosis
Lung transplant
Lung disease due to external agents
Bronchiectasis
Pulmonary congestion and hypostasis
Nonrheumatic tricuspid valve disorders
Nephritis; nephrosis; renal sclerosis
Dependence on respirator [Ventilator] or supplemental oxygen
Postnasal drip
Respiratory failure
Respiratory failure, insufficiency, arrest
Hyperglyceridemia
Voice disturbance
Other diseases of lung
Congestive heart failure (CHF) NOS
Disorders involving the immune mechanism
Congestive heart failure; nonhypertensive
Shortness of breath
Dizziness and giddiness (Light-headedness and vertigo)
Cancer of other lymphoid, histiocytic tissue
Non-Hodgkins lymphoma
Urinary obstruction
p=2.14e-36
p=3.96e-08
p=6.89e-44
p=2.62e-34
p=2.69e-04
p=8.24e-04
p=9.77e-07
p=2.25e-05
p=1.80e-05
p=3.69e-04
p=2.12e-03
p=3.43e-03
p=2.05e-04
p=9.64e-05
p=3.50e-03
p=1.97e-03
p=2.34e-03
p=1.32e-03
p=3.39e-03
p=1.15e-03
p=3.99e-04
p=2.77e-03
p=4.63e-04
p=5.02e-04
p=2.46e-03
Odds Ratio of rs35705950 with SE

Figure S8. PheWAS of *MUC5B* promoter polymorphism rs35705950 in *All of Us*.

A. Phenome-wide association study (PheWAS) Manhattan Plot of phenotype groupings and associations with the *MUC5B* promoter polymorphism rs35705950 in all populations (i.e., all samples). Only phenotypes that passed Bonferroni correction are noted by name.

B. Odds ratios of the top 25 diseases (based on ordered p-values) for associations with rs35705950. Green indicates phenotypes that passed Bonferroni correction. SE = standard error (horizontal bars corresponding to each p-value).
